# The gastrodin biosynthetic pathway in *Pholidota chinensis* Lindl. revealed by transcriptome and metabolome profiling

**DOI:** 10.3389/fpls.2022.1024239

**Published:** 2022-11-03

**Authors:** Baocai Liu, Jingying Chen, Wujun Zhang, Yingzhen Huang, Yunqing Zhao, Seifu Juneidi, Aman Dekebo, Meijuan Wang, Le Shi, Xuebo Hu

**Affiliations:** ^1^ Institute for Medicinal Plants, College of Plant Science and Technology, Huazhong Agricultural University, Wuhan, China; ^2^ Institute of Agricultural Bioresource, Fujian Academy of Agricultural Sciences, Fuzhou, China; ^3^ Innovation Academy of International Traditional Chinese Medicinal Materials, Huazhong Agricultural University, Wuhan, China; ^4^ National-Regional Joint Engineering Research Center in Hubei for Medicinal Plant Breeding and Cultivation, Huazhong Agricultural University, Wuhan, China; ^5^ Medicinal Plant Engineering Research Center of Hubei Province, Huazhong Agricultural University, Wuhan, China; ^6^ Department of Applied Biology, School of Natural Science, Adama Science and Technology University, Adama, Ethiopia; ^7^ Applied Chemistry Department, School of Applied Natural Sciences, Adama Science and Technology University, Adama, Ethiopia; ^8^ Institute of Pharmaceutical Sciences, Adama Science and Technology University, Adama, Ethiopia; ^9^ Shengnongjia Academy of Forestry, Shengnongjia, Hubei, China

**Keywords:** *Pholidota chinensis*, gastrodin, metabolome, transcriptome, molecular mechanism

## Abstract

*Pholidota chinensis* Lindl. is an epiphytic or lithophytic perennial herb of Orchidaceae family used as a garden flower or medicinal plant to treat high blood pressure, dizziness and headache in traditional Chinese medicine. Gastrodin (GAS) is considered as a main bioactive ingredient of this herb but the biosynthetic pathway remains unclear in *P. chinensis*. To elucidate the GAS biosynthesis and identify the related genes in *P. chinensis*, a comprehensive analysis of transcriptome and metabolome of roots, rhizomes, pseudobulbs and leaves were performed by using PacBio SMART, Illumina Hiseq and Ultra Performance Liquid Chromatography Tandem Mass Spectrometry (UPLC-MS/MS). A total of 1,156 metabolites were identified by UPLC-MS/MS, of which 345 differential metabolites were mainly enriched in phenylpropanoid/phenylalanine, flavone and flavonol biosynthesis. The pseudobulbs make up nearly half of the fresh weight of the whole plant, and the GAS content in the pseudobulbs was also the highest in four tissues. Up to 23,105 Unigenes were obtained and 22,029 transcripts were annotated in the transcriptome analysis. Compared to roots, 7,787, 8,376 and 9,146 differentially expressed genes (DEGs) were identified in rhizomes, pseudobulbs and leaves, respectively. And in total, 80 Unigenes encoding eight key enzymes for GAS biosynthesis, were identified. Particularly, glycosyltransferase, the key enzyme of the last step in the GAS biosynthetic pathway had 39 Unigenes candidates, of which, transcript28360/f2p0/1592, was putatively identified as the most likely candidate based on analysis of co-expression, phylogenetic analysis, and homologous searching. The metabolomics and transcriptomics of pseudobulbs versus roots showed that 8,376 DEGs and 345 DEMs had a substantial association based on the Pearson’s correlation. This study notably enriched the metabolomic and transcriptomic data of *P. chinensis*, and it provides valuable information for GAS biosynthesis in the plant.

## Introduction

**Figure 1:**
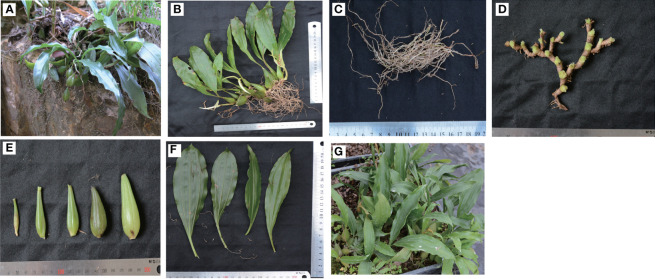
The morphological characteristics and growing environment of *P. chinensis*. **(A)**, Wild growth environment and plants growing on stone surfaces; **(B)**, the whole plant; **(C)**, Roots (designated as B1 for the rest of the sample analysis); **(D)**, Rhizomes (designated as B2 for the rest of the sample analysis); **(E)**, pseudobulbs (designated as B3 for the rest of the sample analysis); and **(F)** leaves (designated as B4 for the rest of the sample analysis) were analyzed; **(G)**, Artificially cultivated plants in a garden.

*Pholidota chinensis* Lindl, a member of the Orchidaceae family, is commonly known as “Shi Xian Tao” in China (**Figures 1A–G**). It is an epiphytic or lithophytic perennial herb widely distributed in southern China (Want et al., 2010; Dunn et al., 2011). The whole plants or pseudobulbs are used as ornamental flowers or folklore medicine in treating high blood pressure, dizziness and headache (Medincine, 2006). It is also orally administered in treating cough, tuberculosis, scrofula, diuresis, and infantile malnutrition as a traditional medicine by the Maonan tribal minorities in Guangxi province of China (Hong et al., 2015). Researchers have shown that polysaccharides, stilbenoids, dihydrophenanthrenes and triterpenoids are the main bioactive components in *P. chinensis* (Yao et al., 2008). These compounds exhibited multiple therapeutic activities including anti-tumor (Luo et al., 2018), anti-oxidant (Yang et al., 2016), anti-bacterial (Ti et al., 2020), anti-diabetic (Ren et al., 2020), anti-inflammatory (Wang et al., 2006), anti-pain and inhibit central nervous system (Liu et al., 2007; Rueda et al., 2014; Wang et al., 2016). Extensive chemical and pharmacological studies have laid a solid foundation for further application of these ingredients as medicine (Yang et al., 2016; Ti et al., 2020).

A Chinese patented medicine “Toutongding Syrup” is made up of *P. chinensis* for treating neurological headaches and concussion sequelae, and the gastrodin (4-hydroxymethylphenyl-β-Dglucopyranoside, GAS) and gastrodigenin (4-hydroxybenzyl alcohol, HBA) were shown to be the primary active ingredients (Weng, 2006; Zou et al., 2017). According to the established high performance liquid chromatography (HPLC) fingerprinting of *P. chinensis*, GAS was one of its analytical markers and its content in *P. chinensis* was higher than another traditional Chinese medicine *Gastrodia elata* Blume. (Zhang et al., 2019; Zhang et al., 2020). *G. elata* is the major source of GAS and HBA that is widely used to treat neurological disorders for centuries in China (Yuan et al., 2018; Zhang et al., 2019; Bae et al., 2022).

The GAS, a phenolic glycoside, is widely used to treat sedative, hypnotic, anticonvulsive and neuroprotective diseases in clinics (Liu and Yang, 2022). Synthesis of GAS is accomplished through glycosylation by a glycosyltransferase (GT) which transforms HBA with different glucose donors (Bai et al., 2016). Toluene was considered as the biosynthetic precursor for HBA that catalyzed by monooxygenase of cytochrome P450 (Tsai et al., 2016). The biosynthetic pathway of phenolic components, including GAS and HBA, were synthesized from phenylalanine through the phenylpropanoid pathway, which was speculated in *G. elata* by transcriptome analysis (Shan et al., 2021), and the biosynthetic pathway of 4-hydroxylbenzaldehyde and vanillin had been well studied in *Vanilla* spp. (Gallage et al., 2014; Wang et al., 2018). Interestingly, whether the precursor is toluene or phenylalanine, the last step is a GT that catalyzes the conversion of HBA to GAS in the GAS biosynthetic pathway (Tsai et al., 2016; Yin et al., 2020). However, the full, native biosynthetic pathway of GAS in *P. chinensis* has still not yet been documented.

To date, the transcriptome and metabolome studies provide effective strategies for understanding the molecular mechanisms of active ingredient formation (Hu et al., 2021; Chen et al., 2022). The combination of transcriptome and metabolome makes it possible to identify genes in any complex biological process with high sensitivity and accuracy (Song et al., 2022). The next-generation sequencing merges short reads into longer fragments by computation and it unavoidably affects the accuracy and integrity in fragments assembly (Cheng et al., 2021). In contrast, the third-generation sequencing technology has an advantage of sequencing reads as long as 100 kb but with lower sequencing accuracy (Liu et al., 2022). Therefore, the combination of next-generation sequencing and third-generation sequencing may assist to make up the shortcomings of each sequencing tool.

In the present study, based on multi-omics comparison, the GAS biosynthesis pathway and the genes involved in *P. chinensis* were elucidated. To the best of our knowledge, this study is the first to dissect the genes for GAS biosynthesis in *P. chinensis* and the same genus.

## Materials and methods

### Plant materials

*P. chinensis* was collected in June 2018 from Lingxia Village, Dongzhang Town, Fuqing City, Fujian Province of China (with 25°41.221´ N; 119°08.358´E and altitude 259 m). The plant sample was authenticated by Prof. Xuebo Hu (College of Plant Science and Technology, Huazhong Agricultural University, Wuhan, China), and Prof. Jingying Chen (Institute of Agricultural Bioresource Fujian Academy of Agricultural Sciences Fuzhou, China). The samples were collected from a wild forest **(** **Figures 1A, B** **),** and the plant part subjected to study was immediately separated into roots (B1, **Figure 1C** **)**, rhizomes (B2, **Figure 1D** **)**, pseudobulbs (B3, **Figure 1E** **)** and leaves (B4, **Figure 1F** **)**. The samples with six independent biological replicates were washed clean, surface dried, and flash-frozen in liquid nitrogen, and then stored at -80°C until chemical composition analysis and RNA extraction. The rest plants were relocated to a greenhouse **(** **Figure 1G** **).**

### UPLC-MS/MS conditions

Liquid chromatography-mass spectrometry (LC-MS) was used to analyze phytochemical constitutes *of P. chinensi*s. The fresh materials of roots, rhizomes, pseudobulbs and leaves (0.1 g) was ground and extracted with 0.5 ml 80% (v/v) MeOH (LC-MS Grade, Thermo Fisher, USA). Samples were sonicated with a Vortex (Kylin-Bell, Jiangsu, China) and centrifuged for 20 min at 15, 000 g. The obtained supernatant was filtered through a 0.22 µm organic nylon needle filter (SCAA-104, ANPEL, Shanghai, China) and stored in a sample bottle (Want et al., 2010; Dunn et al., 2011). The extraction was performed in 6 replicates for statistical analysis. The metabolites were extracted and identified by the Novogene Bioinformatics Technology Co., Ltd.

### Metabolite identification and quantification

The raw data of the mass spectrometry detection were imported into Compound Discoverer 3.1 (CD) software (Hao et al., 2018), used for extraction of metabolite feature. The characteristics of metabolites were obtained based on simple screening of data with their retention time, mass-to-charge ratio and peak alignment, molecular weight of the compound, and the mass deviation and adduct ion information. By matching fragment ions, collision energy and other information of each compound in the mzCloud database, the metabolites in the biological system were identified. Then, the QC Compounds with a CV (Coefficient of Variance) value less than 30% (Dai et al., 2017) were selected and used for final identification. Data quality control was performed to ensure the accuracy and reliability of the data. These metabolites were annotated using the Kyoto encyclopedia of genes and genomes (KEGG) database (http://www.genome.jp/kegg/), human metabolome database (HMDB) (http://www.hmdb.ca/) and Lipidmaps database (http://www.lipidmaps.org/). Principal components analysis (PCA) and Partial least squares discriminant analysis (PLS-DA) were performed with metaX (Wen et al., 2017). Metabolites with significant differences in content were identified according to the thresholds of variable importance in projection (VIP) >1, fold change >2 or <0.5 and P value <0.05. Hierarchical clustering (HCA) and metabolite correlation analysis to reveal the relationship among the samples and metabolites (Chen et al., 2015). The metabolic pathway enrichment of differential metabolites (DEMs) was performed, when the ratio x/n > y/N (x, number of differential metabolites associated with this pathway; y, number of background (all) metabolites associated with this pathway; n: number of differential metabolites annotated by KEGG; N, number of KEGG-annotated background (all) metabolites), metabolic pathway was considered as enriched. When the P-value of metabolic pathway < 0.05, metabolic pathway was considered as statistically significant enrichment.

### HPLC analyses of GAS and HBA

The major constituents of *P. chinensi*s, GAS and HBA, were analyzed by HPLC system. GAS and HBA were extracted from dried and fresh *P. chinensi*s tissues (roots and rhizomes, pseudobulbs as well as leaves) and measured, as described previously (Zhang et al., 2019), with slight modifications. Briefly, dried (0.5 g) and fresh powder of each tissue was extracted in 25 mL of 50% (v/v) methanol by ultrasonication for 30 min. Using the following chromatographic conditions, injection volume, 10 uL; column, Agilent SB-aq (5 µm, 4.6 mm × 250 mm); temperature, 30°C; flow rate, 1.0 mL min^–1^; detector and UV-VIS detector at 220 nm. The mobile phases were containing 99.9% acetonitrile (A) and 0.05% phosphoric acid (B).

### RNA extraction and Illumina sequencing

Frozen tissues were transferred to a mortar pre-cooled by liquid nitrogen and ground with a pestle. Total RNA was extracted from roots, rhizomes, pseudobulbs and leaves (4 tissues× 3 biological replications) by using the RNAprep Pure Plant Kit 264 (Tiangen, Beijing, China), following the manufacturer’s instructions. The quality and quantity of RNA was checked by agarose gel electrophoresis and spectrophotometry (IMPLEN, CA, USA) and Agilent Bioanalyzer 2100 system (Agilent Technologies, CA, USA), respectively. The RNA samples with A260/A280 of 1.8-2.2 were selected for cDNA synthesis.

An Illumina Hiseq platform was conducted using the NGS sequencing. The sequencing libraries were generated using NEBNext^®^ Ultra™ RNA Library Prep Kit for Illumina^®^ (NEB, USA) following manufacturer’s recommendations, and index codes were added to attribute sequences to each sample. The RNA-seq experiment was performed at Novogene Bioinformatics Technology Co., Ltd. The raw data were uploaded to Sequence Read Archive (http://www.ncbi.nlm.-nih.gov/) as accession PRJNA841044.

### RNA extraction and PacBio ISO-Seq

To obtain a complete information of all transcripts, the full-length transcriptome sequencing was adopted in the present study. In order to reduce experimental error, the best RNA sample of three replicates was selected from each sample used in Illumina sequencing, and then mixed together in an equal quantity, as one sample, for SMRT sequencing. The Iso-Seq library was prepared according to the Isoform Sequencing protocol (Iso-Seq) using the Clontech SMARTer PCR cDNA Synthesis Kit and the BluePippin Size Selection System protocol as described by Pacific Biosciences (PN 100-092-800-03). The generated cDNA was re-amplified by PCR. A Qubit fluorometer (Life Technologies, Carlsbad, CA, USA) was used to determine fragment size distribution. The quality of the libraries was assessed using the Agilent Bioanalyzer 2100 system. The SMRT sequencing was performed using the Pacific Biosciences’ real time sequencer using C2 sequencing reagents. The RNA-seq experiment was performed at Novogene Bioinformatics Technology Co., Ltd. The raw data were deposited to Sequence Read Archive (http://www.ncbi.nlm.-nih.gov/) with accession. PRJNA806713.

The sequence data were processed using the SMRTlink 5.0 software (https://www.pacb.com/support/software-downloads/). Circular consensus sequence (CCS) was generated from subread BAM files parameters: min_length 200, max_drop_fraction 0.8, no_polish TRUE, min_zscore -9999, min_passes 1, min_predicted_accuracy 0.8, max_length 18000. The CCS.BAM files were output, which were then classified into full length and non-full length reads using pbclassify.py script, ignore polyA false, minSeq Length 200. Non-full length and full-length fasta files produced were then fed into the cluster step, which does isoform-level clustering, followed by final Arrow polishing, hq_quiver_min_accuracy 0.99, bin_by_primer false, bin_size_kb 1, qv_trim_5p 100, qv_trim_3p 30. Additional nucleotide errors in consensus reads were corrected using the Illumina RNA- seq data with the software LoRDEC (Salmela and Rivals, 2014). After all redundancy corrected, the consensus reads were removed by CD-HIT (Fu et al., 2012), and the final consensus isoforms were obtained for the subsequent analysis.

### Functional annotation

Final consensus isoforms were searched used diamond v0.8.36 software against NCBI non-redundant (Nr), Swiss-Prot, euKaryotic Ortholog Groups (KOG)/Cluster of Orthologous Groups and Kyoto Encyclopedia of Genes and Genomes (KEGG) databases with an E value threshold of 1e^-5^. The BLAST software with E-value ≤1e−5 was used for NT database analysis. The Hmmscan procedure was used in the Pfam database, and GO function categories were performed by Blast2 GO v2.5 based on Pfam annotation. We use the confidence protein sequences of R. ferrugineus or closely related species for ANGLE training, and then run the ANGLE predictions for given sequences (Shimzu et al., 2006). Transcription factors (TF) were performed by the iTAK software (Zheng et al., 2016). Coding Potential Calculator (CPC) (Kang et al., 2017), and Pfam-scan (Finn et al., 2016) to predict the coding potential of transcripts.

### RNA-seq read mapping and expression analysis

The consensus after de-redundancy correction was used the reference sequence (ref), and the clean reads of each sample obtained by Illumina sequencing were aligned to the ref using RSEM software (Li and Dewey, 2011). Further, RSEM software was used to count the comparison results of bowtie2, obtained the read count value of each sample compared to each gene, performed reads per kilo base of transcript per million mapped reads (FPKM) normalization, and then analyzed the expression level of the gene.

### Differential expression analysis

Differential expression analysis of two conditions/groups was performed using the DESeq R package (Love et al., 2014). The DESeq provide statistical routines for determining differential expression in digital gene expression data using a model based on the negative binomial distribution. The resulting P-values were adjusted using the Benjamini and Hochberg’s approach for controlling the false discovery rate. Genes with an adjusted P-value <0.05 found by DESeq were assigned as differentially expressed. Gene Ontology (GO) enrichment analysis of differentially expressed genes or lncRNA target genes were implemented by the GOseq R package (http://www.bioconductor.org/packages/release/bioc/html/goseq.html), in which gene length bias was corrected. The KOBAS software (http://kobas.cbi.pku.edu.cn/download.php) was used to test the statistical enrichment of differentially expressed genes or lncRNA target genes in KEGG pathways.

### Identification of candidate genes involved in the GAS biosynthesis pathway

Candidate genes belonging to the GAS biosynthetic pathway in *P. chinensis* were manually identified according to the annotated sequences in the above databases. Protein coding sequences (CDS) were acquired by Angel software (Shimzu et al., 2006), and multiple sequence alignment was carried out by MEGA7.0 (Kumar et al., 2016).

### Phylogenetic analysis

Amino acid alignments were performed using Clustal W, and phylogenetic trees were built using MEGA7.0 (Kumar et al., 2016), employing the neighbor joining method with 1000 bootstrap replicates, and applying the default settings for other parameters. The GenBank accession numbers/transcript numbers for all sequences are shown in **Supplementary Table S1**.

### Quantitative Real-Time PCR validation

To verify the accuracy of transcriptomic data, 6 DEGs in roots, rhizomes, pseudobulbs and leaves were selected for qRT-PCR verification. Primers were designed using Primer-BLAST on the NCBI website (**Supplementary Table S2**). RNA was reverse transcribed using a TransScript^®^ RT/R1 reagent kit according to the manufacturer’s instructions. The qRT-PCR was performed on an ABI QuantStudio 3. There were three biological and three technical replicates for each sample. The qRT-PCR reaction system (20 μL) consisted of 10 μL of Universal SYBR Green Fast qPCR Mix SYBR Green Master Mix, 1 μL of cDNA, 0.4 μL each of forward and reverse primer, and 8.2 μL of sterile water. The qRT-PCR procedure included 3 min of initiation, followed by 40 cycles at 95°C for 5 s, 60°C for 30 s, and 72°C for 12 s. Relative expression levels were calculated using the 2^–ΔΔCt^ method and normalized according to the actin gene of β-tubulin.

## Results

### Tissue specific metabolites analysis

**Figure 2:**
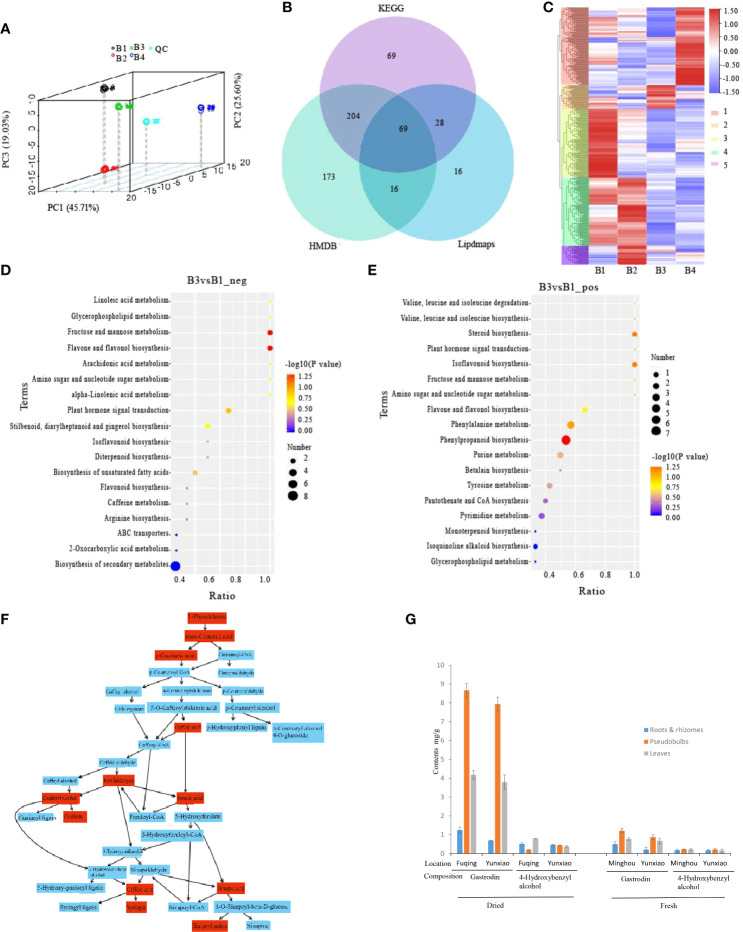
Metabolomes and differential expression of metabolomes (DEMs) of different tissues in *P. chinensis* by UPLC-MS analysis. **(A)**, PCA analysis of all samples. Scattered dots in different colors represent samples from different experimental groups; **(B)**, Venn diagram of annotations in KEGG, HMDB and Lipdmaps database; **(C)**, DEMs clustering heatmap of roots, rhizomes, pseudobulbs and leaves, and divided into five clusters in different color on the left heatmap and marked 1, 2, 3, 4, 5 on the right heatmap. Expression value was calculated based on Log2 Fold change. **(D)**, DEMs of B3 vs B1 on negative ion mode in KEGG pathway enrichment; **(E)**, DEMs of B3 vs B1 on positive ion mode in KEGG pathway enrichment; **(F)**, Phenylpropanoid biosynthesis by Metaboanalyst on line analyses. Red boxes were detected and annotated KEGG components; **(G)**, Gastrodin and 4-Hydroxybenzyl alcohol contents of different tissues by HPLC in dry and fresh *P. chinensis*. PCA, Principal component analysis; KEGG, Kyoto Encyclopedia of Genes and Genomes; Roots (B1), rhizomes (B2), pseudobulbs (B3) and leaves (B4).

To explore the metabolite differences in roots, rhizomes, pseudobulbs and leaves of fresh *P. chinensis*, the samples were analyzed by UPLC-MS/MS **(** **Figure 2A** **)**. A total of 1,156 (positive: 711, negative: 445) metabolites were identified **(** **Supplementary Table S3** **)**. They were subsequently annotated in the KEGG, HMDB and Lipdmaps database, and annotations of 375, 462, and 129 metabolites were obtained, respectively **(** **Figure 2B** **)**. The results of HCA showed that the DEMs were significantly varied in different organs, which were divided into five clusters **(** **Figure 2C** **).** The metabolites were comparable in leaves and pseudobulbs as well as roots and rhizomes. The relative content of GAS, Com_2638 in negative metabolites, was the highest in B3 and the lowest in B1 (**Supplementary Table S4**). Therefore, the comparison group of B3 and B1 was profiled and the results showed (**Figures 2D, E** **)** that 345 DEMs were mainly enriched in phenylpropanoid biosynthesis and phenylalanine metabolism (positive ion model), secondary metabolite biosynthetic process and flavone and flavonol biosynthesis (negative ion model). Phenylpropanoid biosynthesis, including C00079 (L-Phenylalanine), C00423 (trans-Cinnamate), C00811 (4-Coumarate), C01197 (Caffeate), C02666 (Coniferyl aldehyde), C00590 (Coniferyl alcohol), C00761 (Coniferin), C01494 (Ferulate), C02325 (Sinapyl alcohol), C01533 (Syringin), C00482 (Sinapate) and C02887 (Sinapoyl malate), were shown by Metaboanalyst on line analyses (**Figure 2F** **)**. Phenylalanine or its derivatives may be precursors for the biosynthesis of GAS biosynthesis in *P. chinensis.*

### GAS and HBA contents

To investigate GAS and HBA contents in roots, rhizomes, pseudobulbs and leaves, crude MeOH extracts of dried or fresh *P. chinensis* samples from different sites were analyzed by HPLC. The results indicated that both in dried and fresh samples, the GAS of pseudobulbs was the highest (of 0.867 and 0.794% in dried samples, of 0.121 and 0.087% in fresh samples), followed by leaves. The lowest content was detected in roots and rhizomes **(** **Figure 2G** **)**. However, HBA did not show significant difference among sampled plant organs. This result was consistent with the result of UPLC-MS/MS.

### Sequencing and analysis of RNA-Seq

To obtain the transcriptome expression profiles in *P. chinensis*, the RNA was extracted from roots, rhizomes, pseudobulbs and leaves, and mixed together in an equal quantity, as one sample for PacBio Sequel sequencing. As a result, 26.66 Gigabytes Polymerase Read Bases from PacBio Sequel were produced. A total of 506,905 circular consensus sequences (CCS) with an average length of 2,195 bp was obtained after filtration with full passes ≥ 1 and quality > 0.90 **(** **Table 1** **)**. To further improve the accuracy, >6 Gb of raw reads were obtained for each sample from Illumina Hiseq platform performed using NGS sequencing **(** **Supplementary Table S5** **)**. The redundant and similar sequences were removed using CD-HIT software. Finally, 23,105 Unigenes were obtained with an average length of 2,186 bp. It was taken as the reference transcriptome **(** **Table 1** and **Supplementary Figure S1** **)**.

**Table 1:** The characteristics of transcriptome sequences of *P. chinensis* by PacBio sequencing and Illumina.

Item	Number	Average length (bp)	N50	Min_Length	Max_Length
Subreads	14573961	1756	2075	51	——
Circular consensus sequences (CCS)	506905	2195	2542	53	14966
Full-Length non-chimeric Read (FLNC)	450366	2074	2431	56	14705
Polished consensus reads	45157	2054	2388	65	8171
Transcripts of after Illumina correction	45157	2054	2388	65	8171
Unigenes of after CD-HIT De-redundancy	23105	2186	2525	109	8171

**Figure 3:**
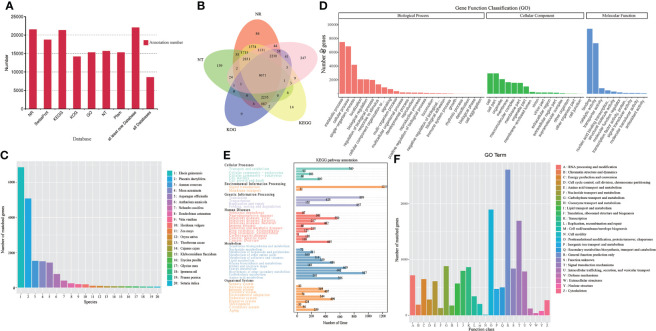
Transcripts functional annotation of *P. chinensis* in NR, NT, Pfam, KOG/COG, Swiss-prot, KEGG, GO databases and analysis. **(A)**: Statistics of the transcripts annotated in different databases. **(B)**: Venn diagram of annotations in NR, GO, KEGG, KOG, and NT databases. **(C)**: Distribution of the top 20 species with matched transcripts in the NR database. 1. *Elaeis guineensis*, 2. *Phoenix dactylifera*, 3. *Ananas comosus*, 4. *Musa acuminate*, 5. *Asparagus officinalis*, 6. *Anthurium amnicola*, 7. Nelumbo nucifera, 8. Dendrobium catenatum, 9. Vitis vinifera, 10. Hordeum vulgare, 11. Zea mays, 12. Oryza sativa, 13. Theobroma cacao, 14. Cajanus cajan, 15. Klebsormidium flaccidum, 16. Erycina pusilla, 17. Glycine max. 18. Ipomoea nil, 19. Prunus persica, 20. Setaria italic. **(D)**: Distribution of GO terms for all annotated transcripts in biological process, cellular component, and molecular function. **(E)**: KEGG pathways annotation by all transcripts. **(F)**: KOG categories of the annotation transcripts. NR, Non-Redundant Protein Sequence Database; NT, Nucleotide Sequence Database; Pfam, database of a large collection of protein families; KOG/COG, EuKaryotic Orthologous Groups of proteins/Clusters Orthologous Groups of proteins; Swiss-prot, annotated protein database and as such an absolute requirement in the toolbox of any protein chemist; KEGG, Kyoto Encyclopedia of Genes and Genomes; GO, gene ontology.

A total of 22,029 transcripts were annotated functionally in this analysis by searching against the GO, KEGG, COG/KOG, NT, Pfam, NR, and Swiss-Prot databases with transcripts 15,307 (69.49%), 21,322(96.79%), 14,155 (64.26%), 15,664 (71.11%), 15,307 (69.49%), 21,512 (97.65%), and 18,731 (85.03%), respectively (**Figure 3A** and **Supplementary Table S5**). However, 8,577 (38.94%) transcripts were annotated in all seven databases **(** **Figure 3B** and **Supplementary Table S6** **)**. Based on the homologous sequence alignment with NR database and statistical analysis, *Elaeis guineensis* was the most homology species (6,867 transcripts, 31.92%) (**Figure 3C** and **Supplementary Table S7**). In KEGG database annotation, the transcripts were grouped into six main categories: Cellular processes (1,406 transcripts), Environmental information processing (1,269 transcripts), Genetic information processing (2,336 transcripts), Human diseases (2,668 transcripts), Metabolism (4,795 transcripts), and Organismal systems (2,215 transcripts). In the metabolism of phenylalanine and terpenoid backbone biosynthesis, 58 and 63 transcripts were involved, respectively. (**Figure 3E** and **Supplementary Table S7**). A group of 128 transcripts were matched to phenylpropandoid biosynthesis (ko00940), including: phenylalanine ammonia-lyase (PAL), 4-coumarate-CoA ligase (4CL), trans-cinnamate 4-monooxygenase (CYP73A) (**Supplementary Figure S2** *)*. GO analysis showed that 15,307 transcripts could be classified into three categories: cellular component, molecular function and biological process. However, the GO terms of metabolic process (7,492 transcripts, 48.94%) were the most annotated transcripts in the Biological process (**Figure 3D** and **Supplementary Table S7**). In KOG classifications, the transcripts yielded 26 functional categories **(** **Figure 3F** and **Supplementary Table S7** **)**. Up to 611 transcripts were annotated in amino acid transport and metabolism and 501 transcripts were annotated in secondary metabolites biosynthesis, transport and catabolism. The number of annotated transcripts identified using the NT, Pfam, and Swiss-Prot databases were summarized in **Supplementary Table S7**. These transcripts involved in amino acid metabolism or secondary metabolism might be partially involved in GAS biosynthesis in *P. chinensis.*

### Analysis of differentially expressed genes (DEGs)

**Figure 4:**
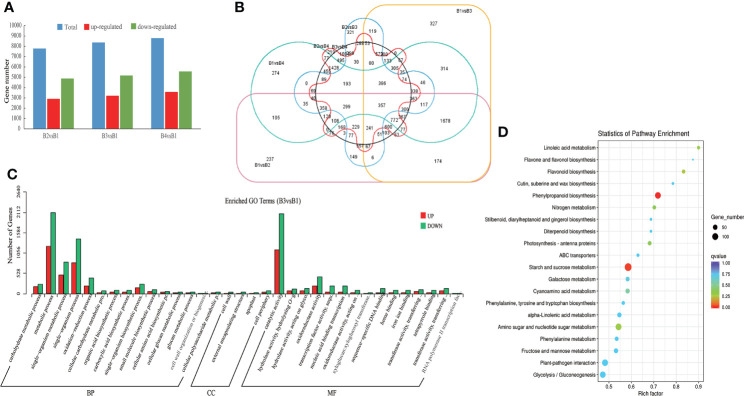
Different expression genes (DEGs) of roots (B1), rhizomes (B2), pseudobulbs (B3) and leaves (B4) in *P. chinensis*. **(A)**, DEGs statistics of B2 vs B1, B3 vs B1, B4 vs B1. The blue bar represents all DEGs, red bar represents up-regulated DEGs, and green bar represents down-regulated DEGs; **(B)**, Venn diagram of DEGs in different comparison groups. The circle color of pink, orange, green, blue, red and black represents B1 vs B2, B1 vs B3, B1 vs B4, B2 vs B3, B2 vs B4 and B3 vs B4, respectively; **(C)**, Enriched GO terms of DEGs in B3 vs B1. The red bar represents up regulation and the blue bar represents down regulation. **(D)**, Enriched KEGG pathway of DEGs in B3 vs B1. The size of the dots represented the number of DEGs. Red and blue represented high and low expression levels, respectively. GO, gene ontology; DEGs, different expression genes.

To identify genes differently expressed in different tissues of *P. chinensis*, 12 cDNA libraries, were mapped to reference sequence (CD-HIT software de-redundant and corrected consensus sequence). The cDNA libraries were generated with mRNA from roots, rhizomes, pseudobulbs and leaves. The matched rate of all the clean reads was >45% (**Supplementary Table S8**). The expression level per sample was shown with read count and FPKM in **Supplementary Table S9**. Compared to roots, the 7,787 DEGs (2,907 up-regulated and 4,880 down-regulated), 8,376 DEGs (3,210 up-regulated and 5,166 down-regulated) and 9,146 DEGs (3,581 up-regulated and 5,565 down-regulated) were identified in rhizomes, pseudobulbs and leaves extracts, respectively (**Figure 4A**). And in total, 16,175 DEGs unigenes in all four tissues were identified. Among different tissues DEGs, only 357 common genes were expressed in all four compared tissues (**Figure 4B**).

To reveal the biological significance of these DEGs, function annotation and enrichment analysis were performed by GO and KEGG database. The analysis of GO functional classification indicated that all the DEGs of B3 and B1 comparison group were grouped into 34 functional groups, including 15 molecular function categories, 15 biological processes, and 4 cellular components. (**Figure 4C** **)**. Metabolic process and single-organism in the biological processes, and catalytic activity in the molecular function were the most enriched terms. However, in almost all terms, down-regulated genes were higher than up-regulated genes. To further illustrate the alterations of gene expression between B3 and B1, the KEGG analysis of all the DEGs of B3 and B1 comparison group was made. The DEGs were enriched in linoleic acid metabolism, flavonoid biosynthesis, phenylpropanoid biosynthesis and others (**Figure 4D**). However, while the up-regulated DEGs of B3 and B1 were mainly enriched in photosynthesis - antenna proteins, phenylpropanoid biosynthesis; the down-regualted DEGs of B3 and B1 were mainly enriched in flavonoid biosynthesis, linoleic acid metabolism and others terms (**Supplementary Figures 3**-**4**.). 28 transcripts were up-regulated in phenylpropanoid biosynthesis of B3 and B1 comparison group, including encoding 4CL, cinnamyl-alcohol dehydrogenase, peroxidase, and beta-glucosidase. These enzymes might be critical for the synthesis of GAS precursors. In addition, the transcripts transcript28360/f2p0/1592, transcript25791/f2p0/1719, etc. had significant expression differences in B3 and B1 comparison group and the *p* value was close to zero.

### The candidate genes involved in GAS biosynthesis pathway

**Figure 5:**
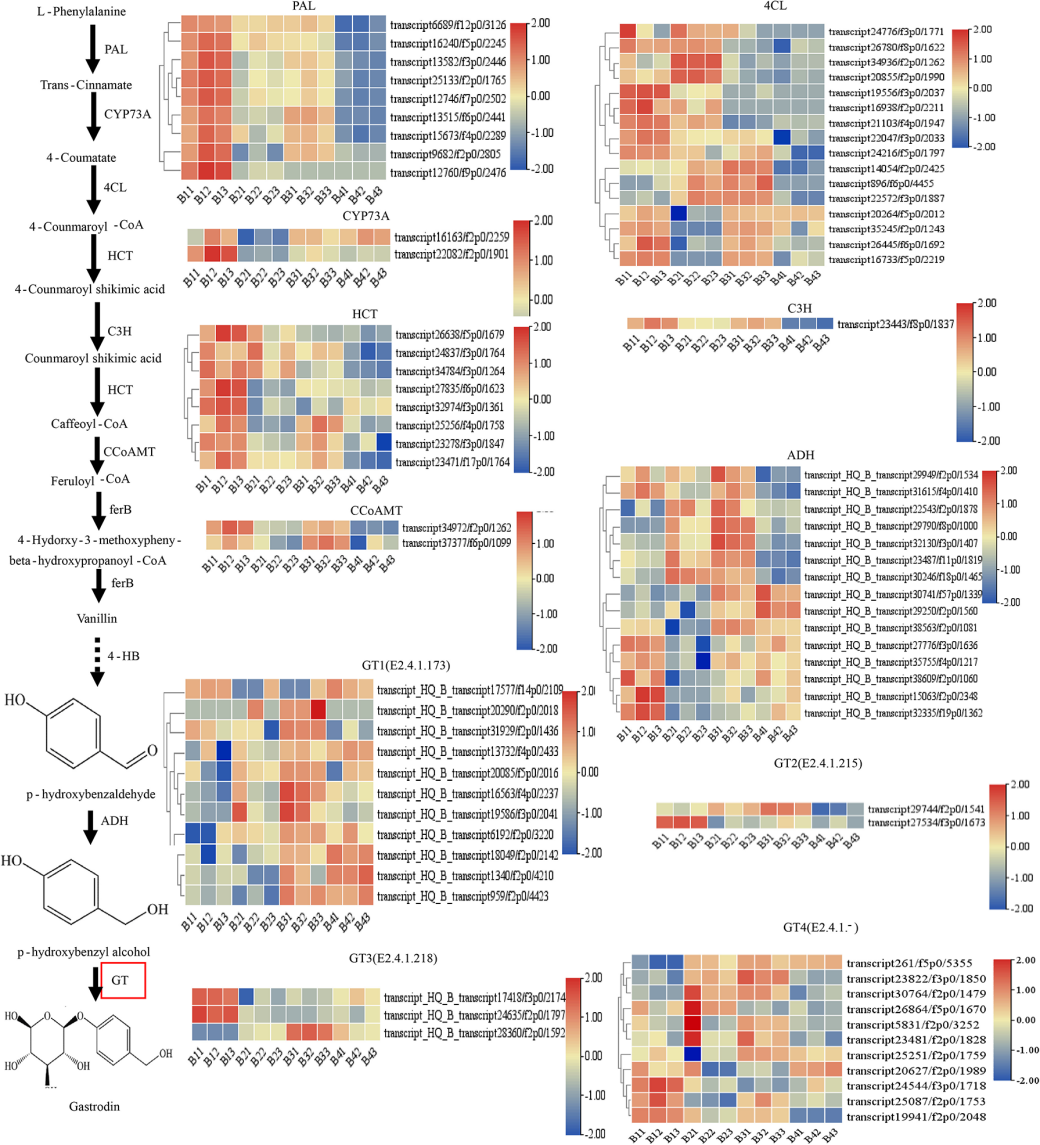
Putative gastrodin biosynthesis pathway in *P. chinensis*. This pathway was constructed based on the KEGG pathway (map00940, map01061) and literature references. The expression levels deduced from the RMPK of each Unigene that encodes the relevant enzyme, were shown as heat map, whereas roots (triplicates as B11, B12, B13), rhizomes (triplicates as B21, B22, B23), pseudobulbs (triplicates as B31, B32, B33) and leaves (triplicates as B41, B42, B43) were separately analyzed. The expression value was calculated based on the Log2 Fold change. Red and blue represented high and low expression levels, respectively. Non-dashed line arrows represent identified enzymatic reactions, and dashed line arrows represent multiple enzymatic reactions through multiple steps.

**Figure 6:**
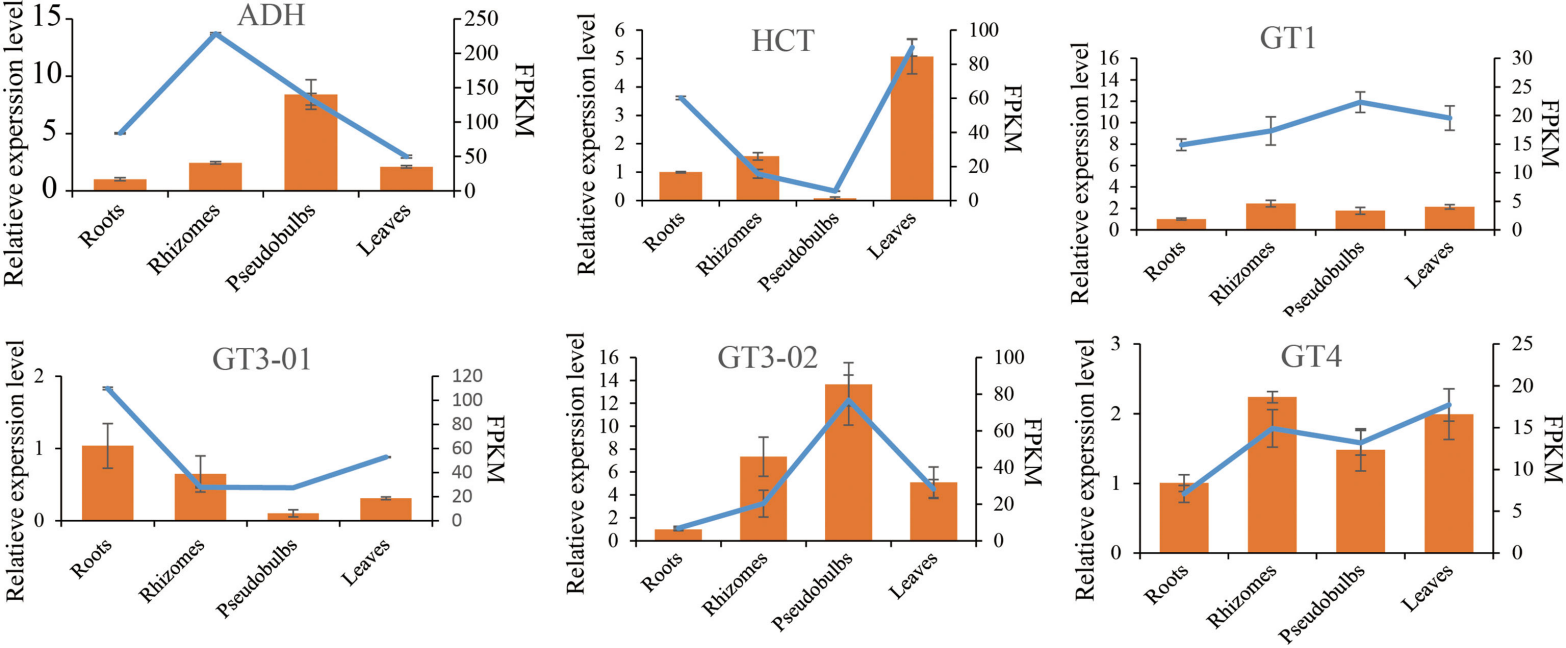
Verification of six selected DEGs by qRT-PCR. Comparison of RNA-seq data (Blue line chart) with qRT-PCR data (Yellow bar graph). The relative qRT-PCR expression level of selected DEGs is shown on the y-axis to the left. *β-tubulin* (TUB) was used as the internal control. Three biological replicates were used. The normalized expression level (FPKM; expected number of Fragments Per Kilobase of transcript sequence per Millions base pairs sequenced) of RNA-seq is indicated on the *y*-axis to the left.

Based on the KEGG pathway (map00940, map00996) analysis as reported in *G. elata* (Shan et al., 2021), the putative GAS biosynthetic pathway of *P. chinensis* is shown in **Figure 6**. The biosynthesis of GAS primarily initiated from the L-phenylalanine, which is derived from the common phenylpropanoid biosynthesis pathway that is broadly distributed in plants (Zhang et al., 2020; Rai et al., 2021). A total of 80 unigenes were identified that encoding eight key enzymes controlling GAS biosynthesis: phenylalanine ammonia-lyase (PAL), trans-cinnamate 4-monooxygenase (CYP73A), 4CL, shikimate O-hydroxycinnamoyltransferase (HCT), 5-O-(4-coumaroyl)-D-quinate 3’-monooxygenase (C3H), caffeoyl coenzyme A-O-methyltransferase (CCoAOMT), alcohol dehydrogenases (ADH) and GT. The relative expression levels of the DEGs in the different tissues were showed in the heatmap (**Figure 5**). However, as the last key enzyme, GT, which catalyzed the GAS synthesis from HBA with UDP-glucose, had 39 Unigenes. These unigenes were divided into four types according to the types of encoded enzymes, including GT1 (3 beta-glucosyltransferase (2.4.1.173)), GT2 (cis-zeatin O-glucosyltransferase (2.4.1.215)), GT3 (hydroquinone glucosyltransferase (2.4.1.218)) and GT4 (others glucosyltransferase (2.4.1-)). Some annotated transcripts GTs were differently expressed in targeted tissues of the studied plant: transcript28360/f2p0/1592, transcript16563/f4p0/2237, transcript19586/f3p0/2041 and transcript25251/f2p0/1759 were highly expressed in pseudobulbs, and lower in leaves, least in roots and rhizomes **(** **Figure 5** **)**.

To verify the accuracy of RNA-seq data, the quantitative real-time PCR (qRT-PCR) was used to validate differential gene expression levels of roots, rhizomes, pseudobulbs and leaves with gene-specific primers (**Supplementary Table S2**). The results showed that the gene relative expression profile was almost consistent with the RNA-seq data. It further demonstrated the credibility of the data generated in the present study (**Figure 6**).

### Identification of glucosyltransferase

**Figure 7:**
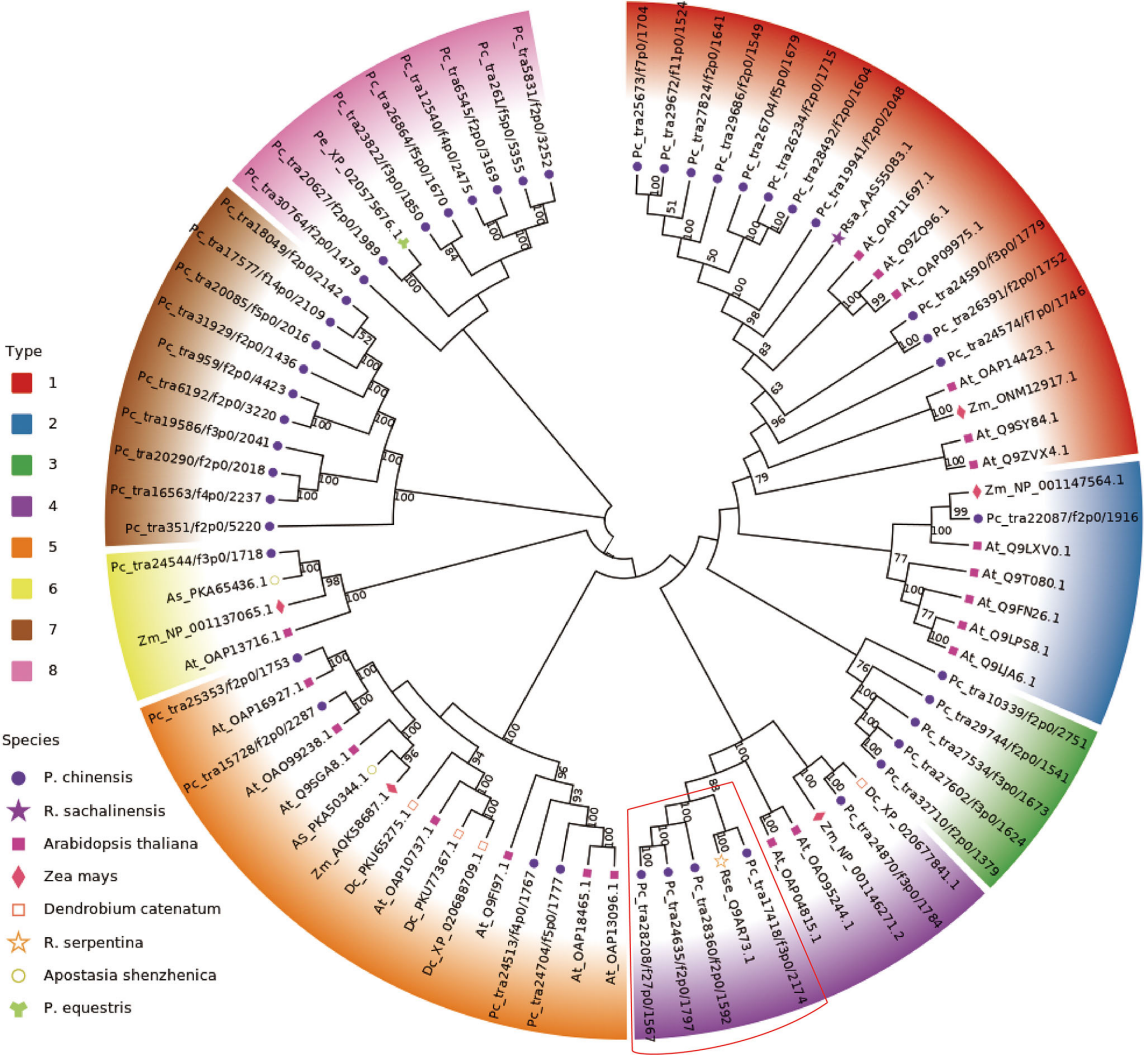
Phylogenetic tree analysis of glucosyltransferase. The constructed phylogenetic tree includes the amino acid sequences of 80 UGT enzymes of 8 species represented by different shapes on the left legends (*Pholidota chinensis, Rhodiola sachalinensis, Arabidopsis thaliana, Zea mays, Dendrobium catenatum, Rauvolfia serpentina serpentine, Apostasia shenzhenica, Phalaenopsis equestris*). The 80 UGT enzymes are clustered into 8 types and displayed with different colors on the left legends and chart. The candidate gene clustering positions obtained in this study are in the red box.

The previous results strongly suggest that the AsUGT, a serpentwood-derived GT convert HBA to GAS with high catalytic efficiency in yeast, compared with UGT73B6 from *Rhodiola sachalinensis* in *Escherichia coli* (Bai et al., 2016; Yin et al., 2020). In *P. chinensis*, significant homology was found using queries from the encoding sequences of the AsUGT and UGT73B6 genes. The most similar transcripts were transcript17418/f3p0/2174 (55.72% identical), transcript28360/f2p0/1592 (47.38% identical), transcript27824/f2p0/1641 (49.90% identical), and transcript29686/f2p0/1549 (48.86% identical) **(** **Supplementary Table S10** **)**. To further characterize the GTs, a phylogenetic tree was constructed based on *P. chinensis* and other plant GT protein sequences, including *Zea mays, Arabidopsis thaliana, Apostasia shenzhenica, Rhodiola sachalinensis, Dendrobium catenatum, Rhodiola sachalinensis* and *Phalaenopsis equestris* (**Supplementary Table S1**). All GT members in *P. chinensis* were divided into eight phylogenetic groups. Among them, AsUGT (Rse_Q9AR73.1), the reference UGT was clustered in the same clade with transcript17418/f3p0/2174, transcript28360/f2p0/1592, transcript24635/f2p0/1797 and transcript28208/f27p0/1567, whereas UGT73B6 (Rsa_AAS55083.1) was clustered in the same clade with transcript19941/f2p0/2048 **(** **Figure 6** **)**. The gene expression level of these transcripts exhibited great tissue-specific tendency. While others were highly expressed in the roots, transcript28360/f2p0/1592 was the only one with high expression in the pseudobulbs. Meanwhile, homologous searching by using queries from the transcript TRINITY_DN50323_c0_g1(which might participate in glucosylation in the GAS biosynthetic pathway of *G. elata*) (Tsai et al., 2016), showed significant homology with transcript20627/f2p0/1989 (84.29% identical) in *P. chinensis* and XM_020720017.1 (83.4% identical) in *P. equestris*, respectively **(** **Supplementary Table S11** **).** However, the expression level of transcript20627/f2p0/1989 was the highest in leaves and lower in pseudobulbs, which might be not the best candidate gene (**Figure 7**).

### Integrative analysis of transcriptomics and metabolomics

**Figure 8:**
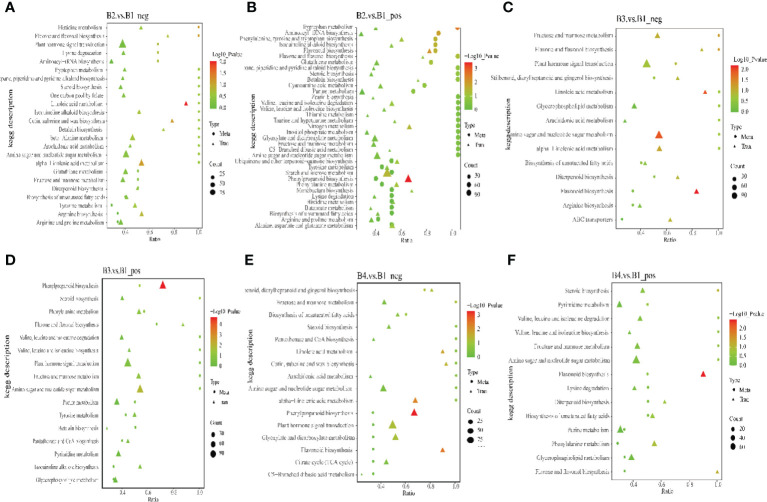
The KEGG enrichment bubble chart of co-expression DEMs and DEGs in B2 vs B1, B3 vs B1 and B4 vs B1 group. Dots represent DEMs. Triangles represent DEGs. Dots or triangles size represent enriched in the pathway number of metabolites or genes. “P value” is the *p* value of the transcription or metabolism pathway enrichment. **(A, C, E)**, negative ion mode, **(B, D, F)**, positive ion mode.

To obtain a deeper understanding, a multi-omics analysis was performed. These analyses integrated the metabolomics with the transcriptomic data. In negative/positive ion mode, top 50 DEMs (sorted by p value from small to large) and top 100 DEGs (sorted by *p* value from small to large) of B2 and B1 comparison group, B3 and B1 comparison group, B4 and B1 comparison group were shown in **Supplementary Figures 5**–**10**. These DEMs and DEGs had a stronger positive or negative connection (R>0.9). To identify the major biochemical pathways and signal transduction involved pathways of DEMs and DEGs, all DEMs and DEGs were matched to the KEGG pathway. The results revealed that the DEMs and DEGs were main enriched in phenylpropanoid biosynthesis and linoleic acid metabolism of B2 and B1 comparison group, phenylpropanoid biosynthesis and amino sugar and nucleotide sugar metabolism of B3 and B1 comparison group, phenylpropanoid biosynthesis and flavonoid biosynthesis of B4 and B1 comparison group (**Figure 8**). The phenylpropanoid biosynthesis may be critical for GAS biosynthesis in *P. chinensis*. There were a notable association (R>0.9) between 8,376 DEGs and 345 DEMs based on the Pearson’s correlation coefficient in B3 and B1 comparison group (**Supplementary Table S12**). And the results showed that no matter in positive or negative ion mode, there were metabolites that had significant correlation with most genes, such as coniferyl aldehyde, butylparaben, 3_4_5-trimethoxycinnamic acid, monobenzyl phthalate etc. Coniferyl aldehyde, a natural non-toxic and anti-inflammatory phenolic compound extracted from edible and medicinal plants (Wang et al., 2020), might be involved in the biosynthesis of GAS (Huccetogullari et al., 2019) and it had significant associations (R>0.9) with 6266 DEGs (**Supplementary Table S13** **)**.

To further understand the relationship between metabolites and genes in common pathway, DEGs and DEMs of B3 and B1 comparison group were simultaneously mapped to the KEGG pathway. The results in negative ion mode showed that 2 DEMs and 42 DEGs (fructose and mannose metabolism), 2 DEMs and 7 DEGs (flavone and flavonol biosynthesis), 2 DEMs and 115 DEGs (plant hormone signal transduction), 2 DEMs and 11 DEGs (stilbenoid, diarylheptanoid and gingerol biosynthesis) enriched the corresponding biological processes (**Figure 8C**). However, the results in positive ion mode showed that 7 DEMs and 92 DEGs, 2 DEMs and 19 DEGs, 4 DEMs and 31 DEGs, 2 DEMs and 7 DEGs were enriched phenylpropanoid biosynthesis, steroid biosynthesis, phenylalanine metabolism, and flavone and flavonol biosynthesis, respectively **(** **Figure 8D** **)**. Furthermore, the putative candidate gene transcript28360/f2p0/1592 in GT3 was significantly negative correlated with coniferylaldehyde and spermidine, but had a significantly positive correlation with GAS, isoeugenol and sinapoyl malate (**Supplementary Table S14** **)**. These results are consistent with transcriptome or metabolome results.

## Discussion

The GAS is the second compound identified from the plant *G. elata* after vanilyalcohol. It is a phenolic glycoside that chemically known as 4-hydroxybenzyl alcohol-4-*O-*β*-D*-glucopyranoside. And it is also the main bioactive constituent of another TCM *Rhizoma Gastrodiae* (Tao et al., 2009; Liu et al., 2018). Being the largest and the most widespread class of plant secondary metabolites, phenolics have been extensively researched due to the diverse health benefits. Some of these include flavonoids, lignans, coumarins, chalcones, and phenolic acids, which participate in the regulation of plant growth, seed germination, and in defense responses (Acosta-Estrada et al., 2014; Naikoo et al., 2019). The GAS content is the most appreciated analytical marker for the quality standardization of *P. chinensis* (Zhang et al., 2019). The mechanism of GAS action is gradually being understood and recognized (Chen et al., 2022; Yang et al., 2022). Several reports have shown that the content of GAS in *P. chinensis* was higher than the content in *G. elata*, one of the major sources of GAS (Zhang et al., 2019; Wang et al., 2022).

Metabolomics, especially untargeted metabolomics using LC-MS, is considered to be the best omics technique to represent the phenotypes because its data analysis based on the state of biochemical activity in the living organism (Fraisier-Vannier et al., 2020; Zeki et al., 2020). In this study, 1,156 metabolic differences were identified in roots, rhizomes, pseudobulbs and leaves of fresh *P. chinensis* using the UPLC-MS/MS. Many of the metabolites were phenolic compounds that were mainly enriched in phenylpropanoid biosynthesis, flavone and flavonol biosynthesis, and phenylalanine metabolism. And some of these compounds were known to exhibit antioxidant, antidiabetic, and anti-inflammatory activities (Ren et al., 2020). Simultaneously, HPLC analysis revealed that pseudobulbs were the primary tissue of GAS, followed by leaves, roots and rhizomes.

Some medicinal plants lack genomic information due to the wide variety, limiting some research, such as the integrity of transcriptome assembly (Cheng et al., 2021). The combination of third-generation sequencing and second-generation sequencing is an effective method for gene mining without reference genome (Liu et al., 2022). In the present study, the mean length of predicted unigenes (2,186 bp) the N50 length (2,525 bp) were longer than some other traditional medicine including *G. elata* (Tsai et al., 2016), *Dendrobium officinale* (Li et al., 2021) and *Dendrobium sinense* (Zhang et al., 2021), indicated that the transcriptome assembly were of high reliability and quality.

The GAS was synthesized from HBA with UDP-glucose *via* glycosylation catalyzed through GT, and HBA was synthesized from cresols (toluene) degradation through two steps of hydroxylation *via* monooxygenase in *G. elata* (Tsai et al., 2016). The monooxygenase 1.14.13.-, which were reported in *G. elata* (Tsai et al., 2016), were not discovered in *P. chinensis.* However, it was also reported that GAS could be synthesized *via* the phenylpropanoid pathway, and the PAL, C4H, 4CL and GT are the key genes in biosynthetic pathway of GAS in *G. elata* (Shan et al., 2021). It is worth noting that GAS was synthesized from glucose by an artificial microbial pathway with key genes of ADHs and GT in *Saccharomyces cerevisiae* and *Escherichia coli*, respectively (Bai et al., 2016; Yin et al., 2020). Glycosylation is often the last step in the biosynthesis of natural products in plants and plays an important role in a variety of biosynthetic pathways (He et al., 2022). According to the above analysis, although the starting point of GAS synthesis is different, but the last step is same, that is, GT catalyzes the conversion of HBA to GAS. In this study, putatively 80 unigenes involved in the biosynthetic pathway of GAS in *P. chinensis* were identified including genes for PAL, CYP73A, 4CL, HCT, C3H, CCoAOMT, ADH and GT. The GT (39 unigenes) were divided into four subgroups according to the types of encoded enzymes. Among all transcripts being found, transcript28360/f2p0/1592, transcript16563/f4p0/2237, transcript19586/f3p0/2041 and transcript25251/f2p0/1759 were highly expressed in pseudobulbs, and lower in other targeted plant parts (leaves, roots and rhizomes), which could be key candidates. Based on phylogenetic tree analysis, the transcript28360/f2p0/1592 in GT3 was deduced as the best candidate gene because it shares a highly homologous sequence with AsUGT, which was identified as the plant-derived GT that converts HBA to GAS with high catalytic efficiency in yeast (Yin et al., 2020).

Integrated analysis of transcriptome and metabolome provides an efficient approach for the research of metabolic networks and key genes. For example, multi-omics were applied for flavonoid biosynthesis in a purple tea plant cultivar (Song et al., 2022), the response of *Zanthoxylum bungeanum* and apple to different stresses (Li et al., 2021; Sun et al., 2021). It is worth noting that the proteome (Camp et al., 2022) is also often analyzed together with the transcriptome or metabolome, but this study has not yet performed a proteome of *P. chinensis*. Coniferylaldehyde, coniferyl alcohol, isoeugenol and sinapoyl malate were members of dominant group of volatile compounds, and those volatile phenyl propene formation might takes two enzymatic steps with lignin, and they were perhaps involved in the synthesis of GAS precursors (Ramya et al., 2020).

Other than our focus on the dissection of transcriptomic and metabolic profiles of the extremely less studied traditional medicine for understanding of the GAS biosynthetic pathway, our study, as the first in the genus, also provides useful data for other basic researches on *P. chinensis* and related species. For example, the plant undergoes a well differentiated developmental stage of pseudobulb and our data could be lent for mining of the molecular mechanisms of pseudobulb development.

## Conclusion

The GAS is an important active component of a traditional Chinese medicine, but its biosynthetic pathway in *P. chinensis* is still unclear. In the present study, the biosynthetic pathway of GAS in *P. chinensis* was speculated by combination of transcriptome and metabolome analysis. Unigenes involved in the biosynthetic pathways, as well as the metabolites, were identified. Besides commonly known unigenes in the synthetic pathway for PAL, CYP73A, 4CL, HCT, C3H, CCoAOMT, and ADH, best candidates for the last synthetic step of GAS, the transcript28360/f2p0/1592, were assured by bioinformatics. Since the growth of the plant is extremely slow and it is not practical to cultivate it in large area to gain sufficient yield and profit, the biosynthetic pathway disclosed in this study, especially the last unique GT for GAS, will be extremely useful for possible biosynthetic engineering of the chemical in microbial platforms. To the best of our knowledge, this study is the first exploration of the genes involved in the GAS biosynthesis in *P. chinensis* and plants in the same genus.

## Data availability statement

The original contributions presented in the study are publicly available. This data can be found here: NCBI, PRJNA841044 and PRJNA806713.

## Author contributions

XH conceived, supervised, and wrote-reviewed the manuscript. SJ, AD, and JC reviewed the draft. BL wrote and reviewed the draft. BL, WZ, YH, YZ, MW, and LS performed the experiments and carried out the analysis. BL and XH designed the experiments. XH and JC co-founded and co-administrated the project. All authors approved the final version.

## Funding

This research financially supported by Fundamental scientific research projects of non-profit scientific research institutes in Fujian Province (2020R1034003), Collaborative Innovation of the Fujian Provincial People’s Government (XTCXGC2021003), Fujian Provincial Finance Special Project to Science and Technology Innovation Team of Fujian Academy of Agricultural Sciences (CXTD2021014-2), Young Talents in Science and Technology Project of Fujian Academy of Agricultural Sciences (YC2021005). This research is also funded by Shengnongjia Academy of Forestry, Hubei, China (No. SAF202102), Hubei Technology Innovation Center for Agricultural Sciences - ‘2020 key technology research and demonstration project of safe and efficient production of genuine medicinal materials’ (No. 2020-620-000-002-04), China-Bulgaria science and technology exchange meeting for traditional medicine (year of 2020), Pinghu Municipal Bureau of Agriculture and Rural Affairs (PH2020005) to XH.

## Conflict of interest

The authors declare that the research was conducted in the absence of any commercial or financial relationships that could be construed as a potential conflict of interest.

## Publisher’s note

All claims expressed in this article are solely those of the authors and do not necessarily represent those of their affiliated organizations, or those of the publisher, the editors and the reviewers. Any product that may be evaluated in this article, or claim that may be made by its manufacturer, is not guaranteed or endorsed by the publisher.

## Supplementary material

Supplementary Figure 1 Assemble Unigene length distribution of *P. chinensis* by PacBio sequencing and Illumina data correction, Abscissa (length/bp), ordinate (number of genes).

Supplementary Figure 2 The transcripts were matched to phenylpropandoid biosynthesis (ko00940)

Supplementary Figure 3 Up-regulated DEGS of B3 vs B1. The size of the dots indicates the number of DEGS in this pathway, and the colors of the dots correspond to different q value ranges, which is closer zero (red color) and more significant enrichments.

Supplementary Figure 4 Down-regulated DEGS of B3 vs B1. The size of the dots indicates the number of DEGS in this pathway, and the colors of the dots correspond to different q value ranges, which is closer zero (red color) and more significant enrichments.

Supplementary Figure 5 The correlation heat map in negative ion mode of co-expression B2 vs B1 DEMs & B2 vs B1 DEGs.

Supplementary Figure 6 The correlation heat map in positive ion mode of co-expression B2 vs B1 DEMs & B2 vs B1 DEGs.

Supplementary Figure 7 The correlation heat map in negative ion mode of co-expression B3 vs B1 DEMs & B3 vs B1 DEGs.

Supplementary Figure 8 The correlation heat map in positive ion mode of co-expression B3 vs B1 DEMs & B3 vs B1 DEGs.

Supplementary Figure 9 The correlation heat map in negative ion mode of co-expression B4 vs B1 DEMs & B4 vs B1 DEGs.

Supplementary Figure 10 The correlation heat map in positive ion mode of co-expression B4 vs B1 DEMs & B4 vs B1 DEGs.

Supplementary Table 3 Species of components in positive and negative ion modes by UPLC-MS/MS.

Supplementary Table 4 The GAS relative content of different tissue by UPLC-MS/MS.

Supplementary Table 5 Sequencing data quality from Illumina Hiseq.

Supplementary Table 6 Annotation and classification of detected unigenes by the seven datasets.

Supplementary Table 7 The annotation of NR, GO, KEGG, KOG, NT, Pfam, Swissprot.

Supplementary Table 8 The comparison rate of the second-generation data map to the third-generation data.

Supplementary Table 9 The expression level of each sample.

Supplementary Table 10 Based on amino acid blast results in *P. chinensis* with AsUGT and UGT73B6 genes as query.

Supplementary Table 11 Blast results of GT in *P. chinensis* and *Phalaenopsis equestris* with candidate gene TRINITY_DN50323_c0_g1 of *Gastrodia elata* as query.

Supplementary Table 12 The correlation in positive and negative ion mode of DEMs & DEGs based on r^2^>0.91 and p<0.01.

Supplementary Table 13 Coniferyl aldehyde correlation DEGs based on r^2^>0.91 and p<0.01.

Supplementary Table 14 The Correlation putative candidate gene transcript28360/f2p0/1592 in GT3 with metabolites.
