# Supplementary figures and images for: The gastrodin biosynthetic pathway in Pholidota chinensis Lindl. revealed by transcriptome and metabolome profiling
Source: Front Plant Sci. 2022 Nov 3;13:1024239. doi: 10.3389/fpls.2022.1024239 (PMC9673822; doi:10.3389/fpls.2022.1024239)

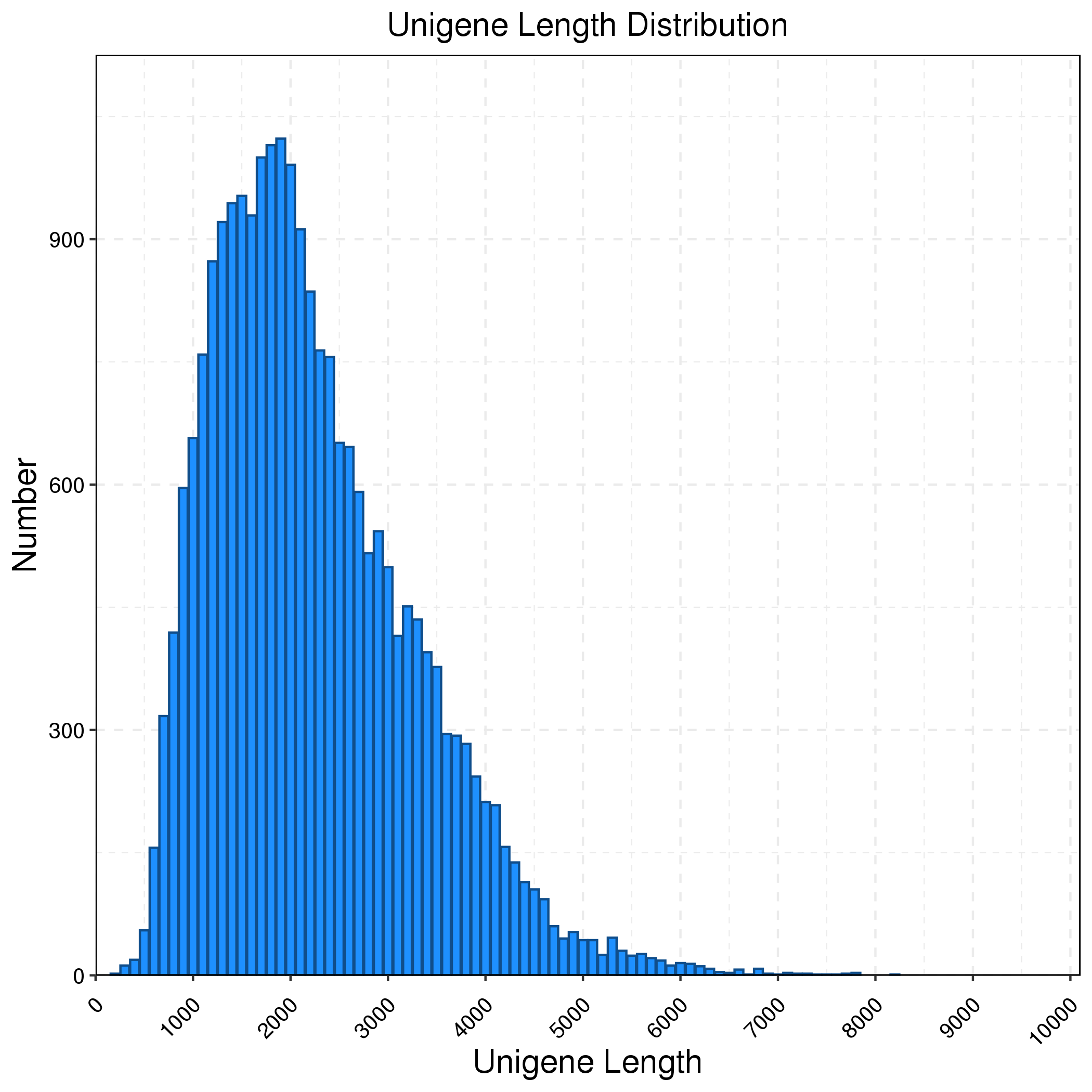

Supplement: Supplementary Figure 1 — Assemble Unigene length distribution of P. chinensis by PacBio sequencing and Illumina data correction, Abscissa (length/bp), ordinate (number of genes). [file Image_1.png]

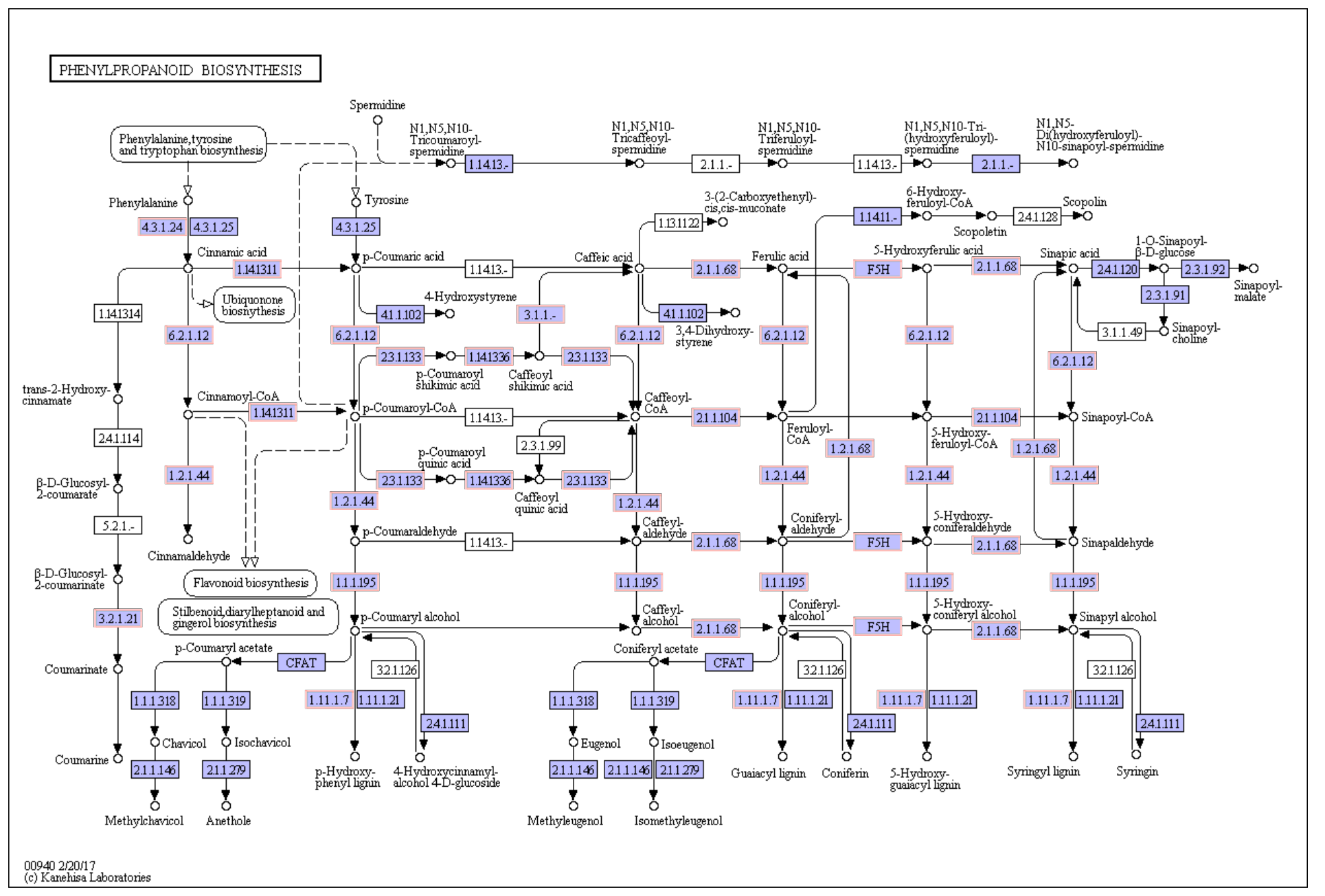

Supplement: Supplementary Figure 2 — The transcripts were matched to phenylpropandoid biosynthesis (ko00940) [file Image_2.png]

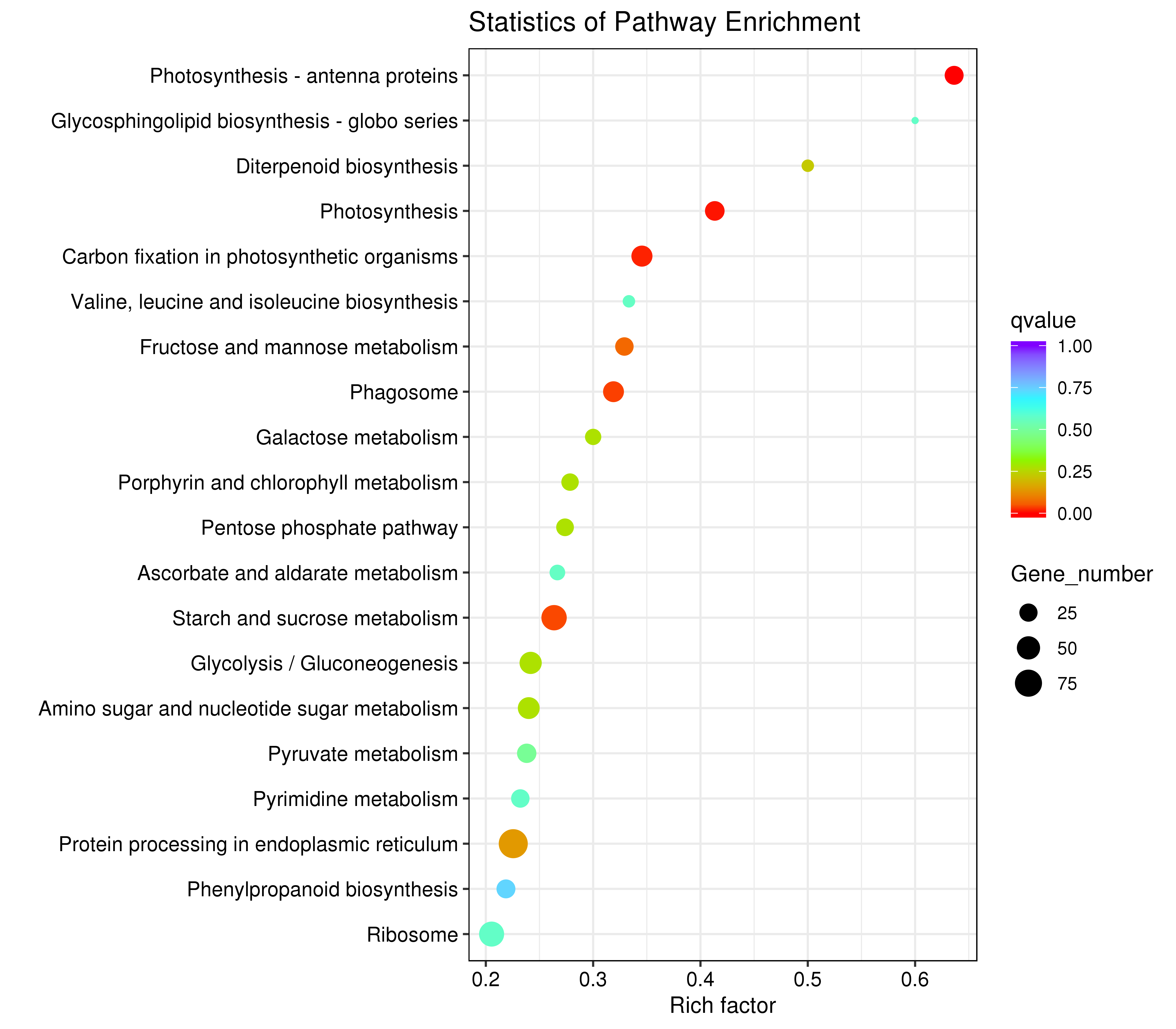

Supplement: Supplementary Figure 3 — Up-regulated DEGS of B3 vs B1. The size of the dots indicates the number of DEGS in this pathway, and the colors of the dots correspond to different q value ranges, which is closer zero (red color) and more significant enrichments. [file Image_3.png]

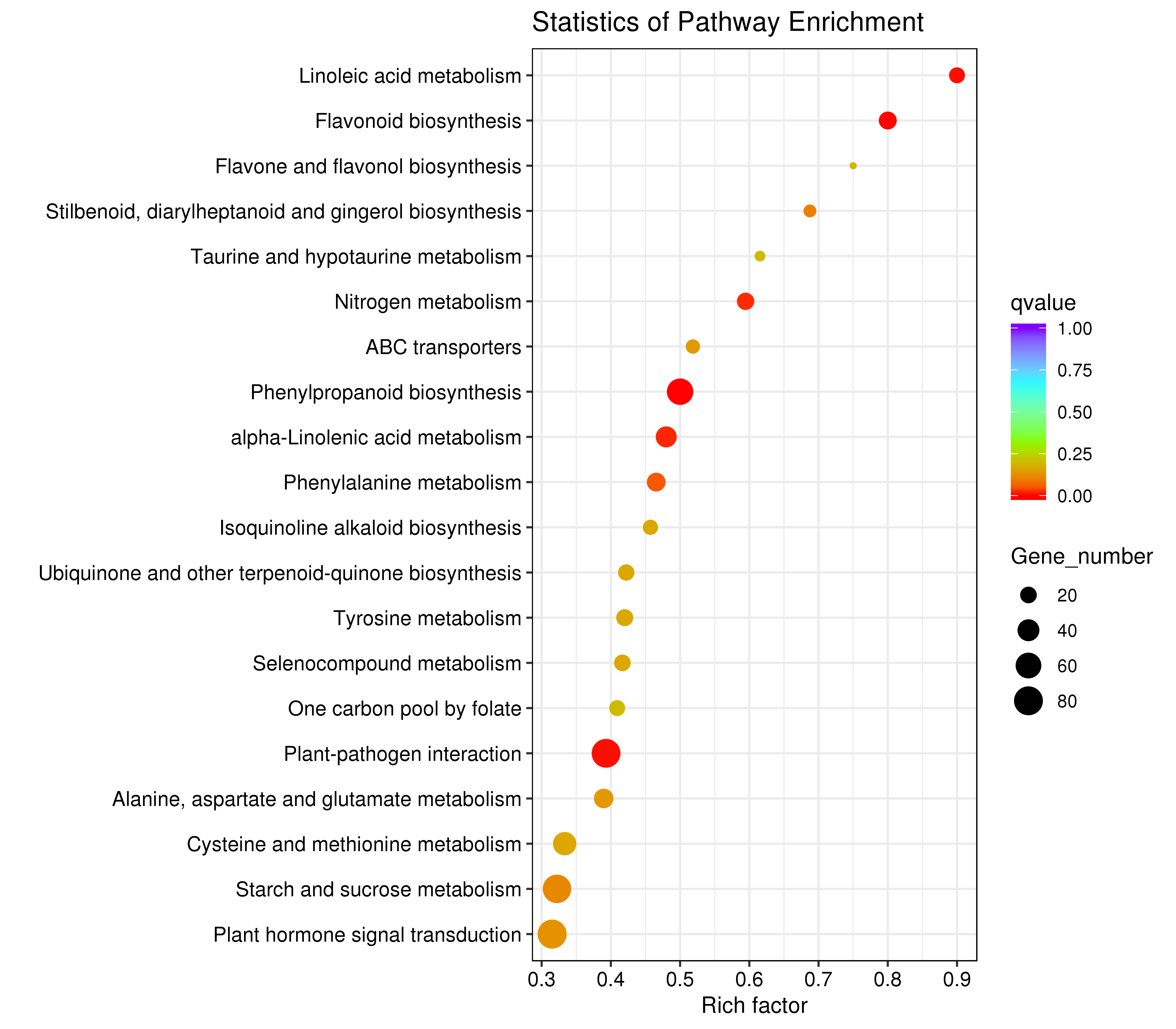

Supplement: Supplementary Figure 4 — Down-regulated DEGS of B3 vs B1. The size of the dots indicates the number of DEGS in this pathway, and the colors of the dots correspond to different q value ranges, which is closer zero (red color) and more significant enrichments. [file Image_4.png]

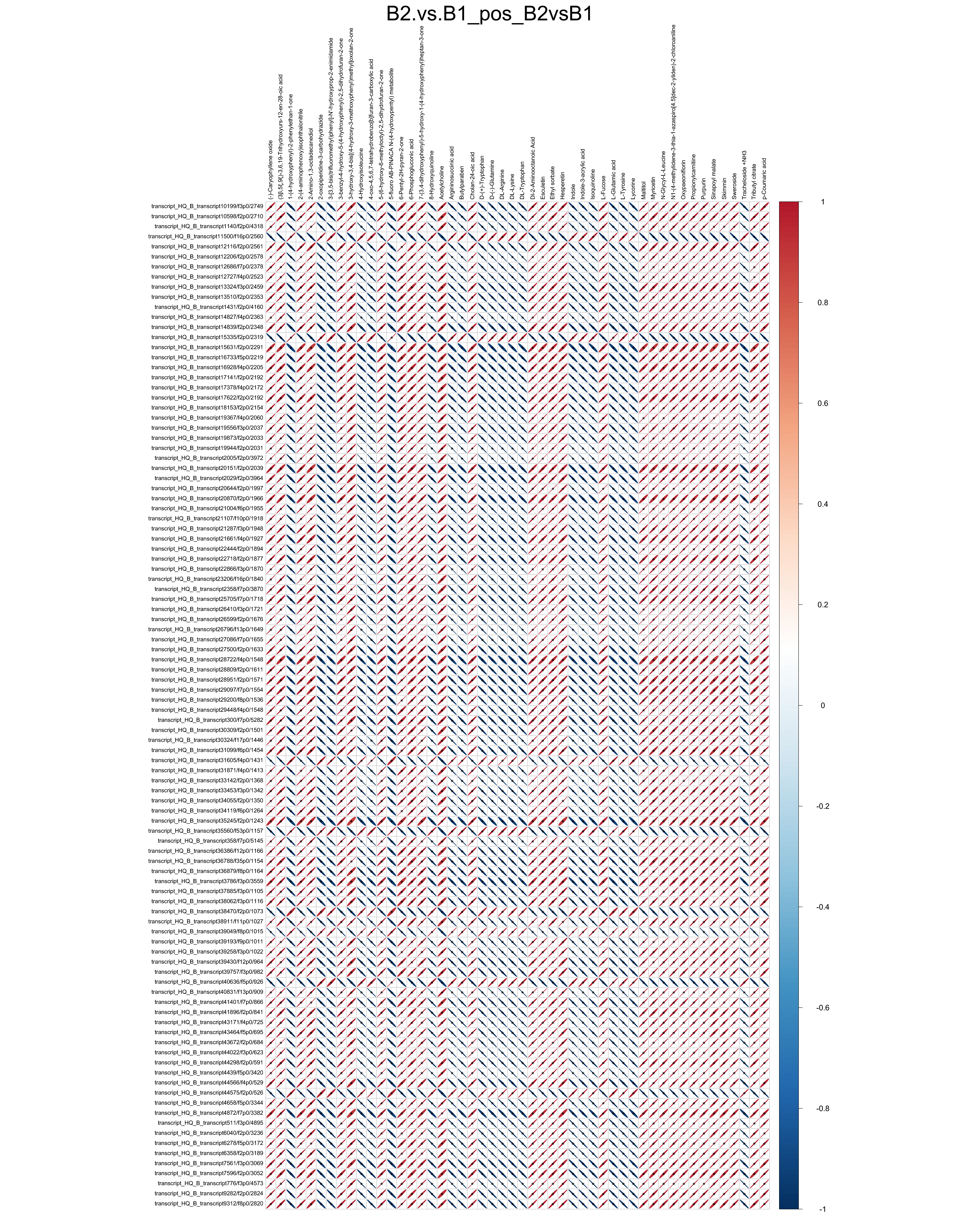

Supplement: Supplementary Figure 5 — The correlation heat map in negative ion mode of co-expression B2 vs B1 DEMs & B2 vs B1 DEGs. [file Image_5.png]

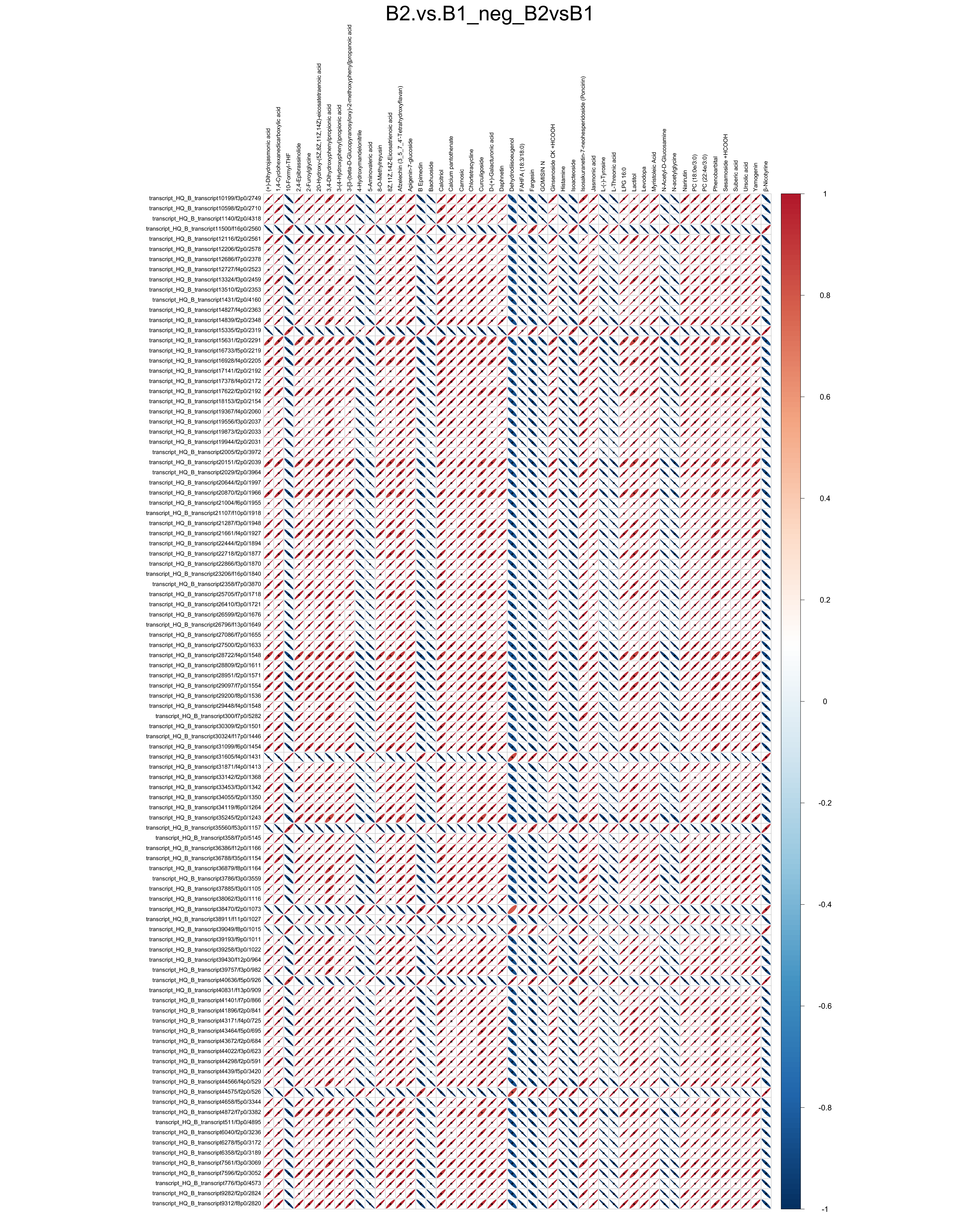

Supplement: Supplementary Figure 6 — The correlation heat map in positive ion mode of co-expression B2 vs B1 DEMs & B2 vs B1 DEGs. [file Image_6.png]

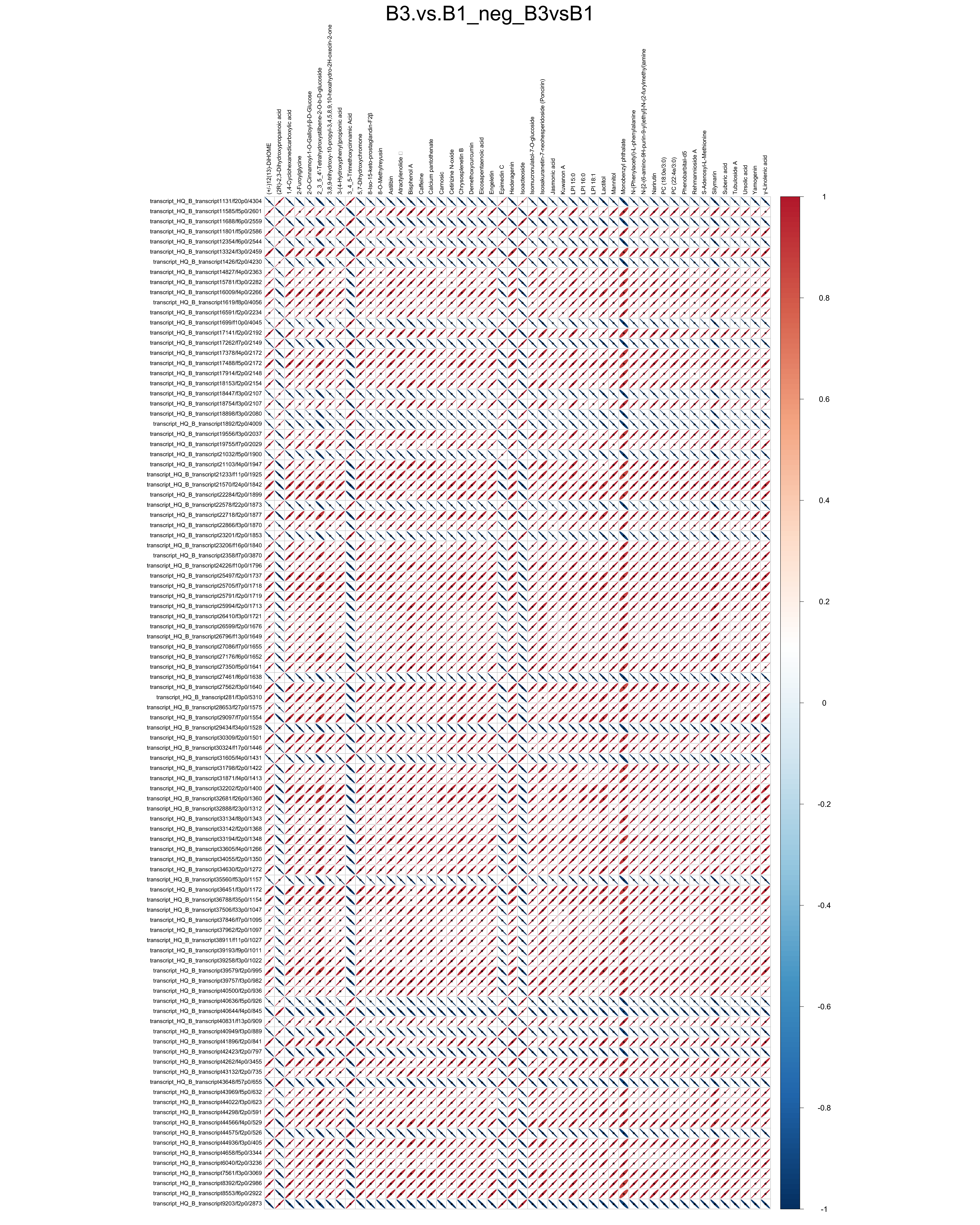

Supplement: Supplementary Figure 7 — The correlation heat map in negative ion mode of co-expression B3 vs B1 DEMs & B3 vs B1 DEGs. [file Image_7.png]

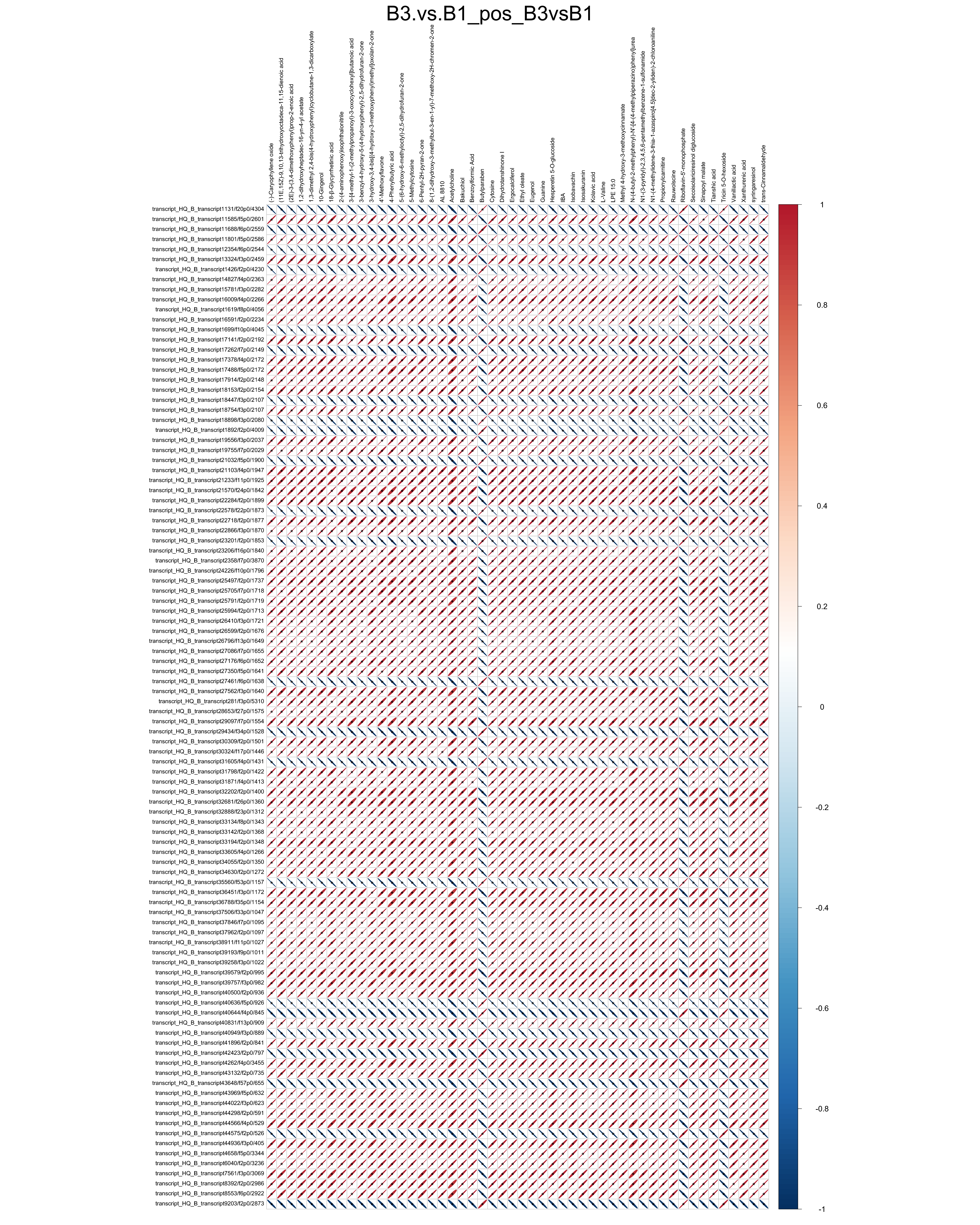

Supplement: Supplementary Figure 8 — The correlation heat map in positive ion mode of co-expression B3 vs B1 DEMs & B3 vs B1 DEGs. [file Image_8.png]

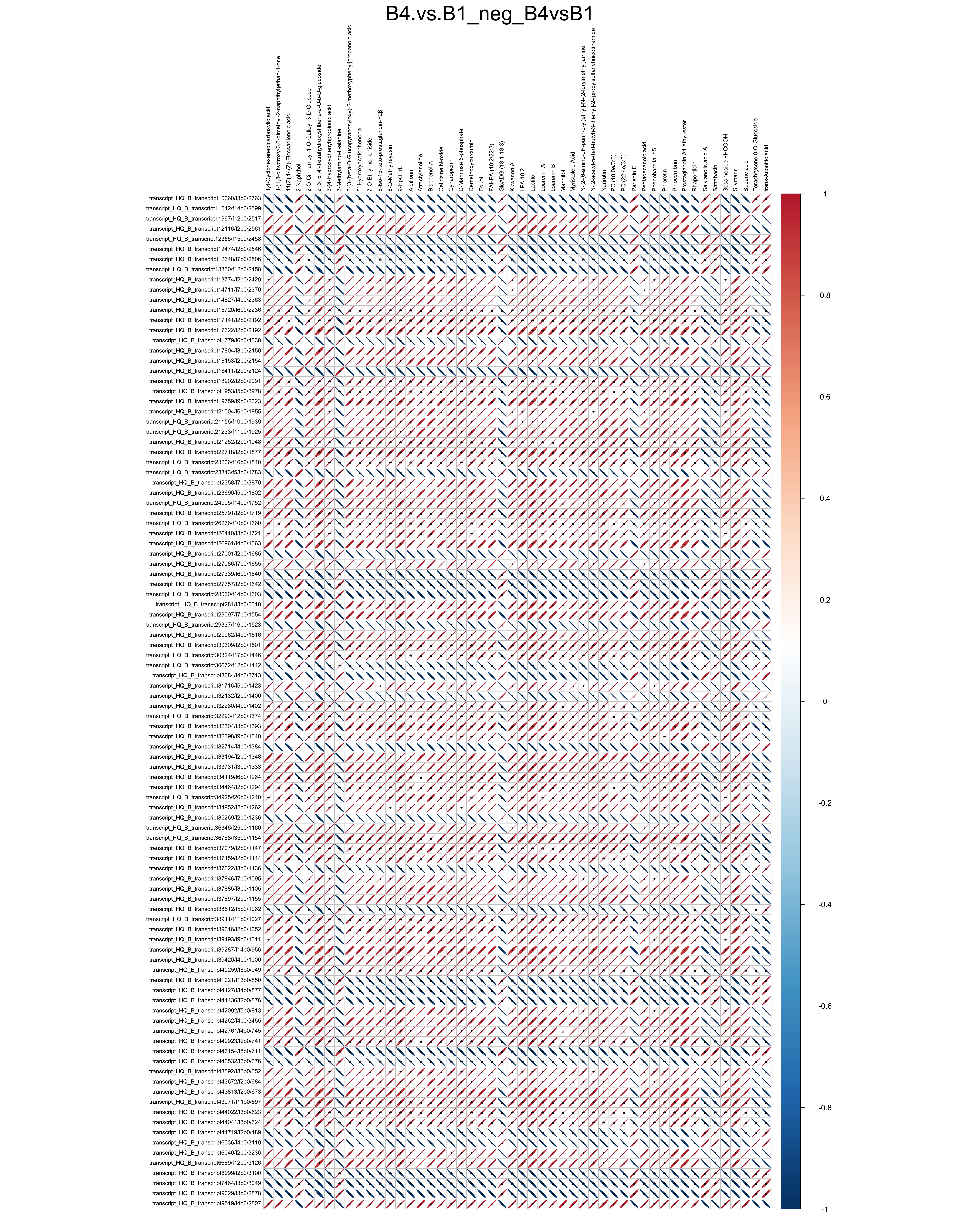

Supplement: Supplementary Figure 9 — The correlation heat map in negative ion mode of co-expression B4 vs B1 DEMs & B4 vs B1 DEGs. [file Image_9.png]

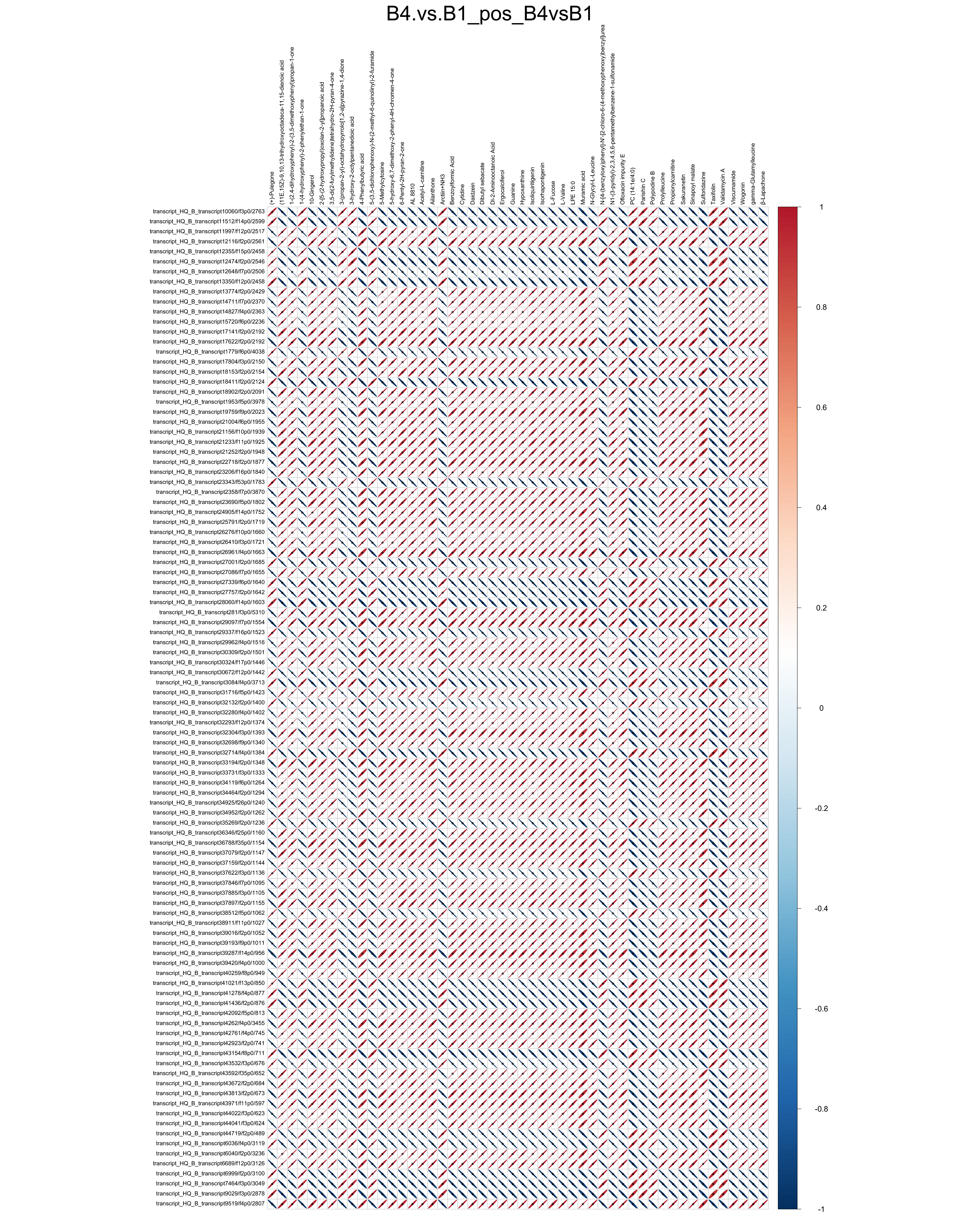

Supplement: Supplementary Figure 10 — The correlation heat map in positive ion mode of co-expression B4 vs B1 DEMs & B4 vs B1 DEGs. [file Image_10.png]
