# Supplementary material for: The gastrodin biosynthetic pathway in Pholidota chinensis Lindl. revealed by transcriptome and metabolome profiling
Source: Front Plant Sci. 2022 Nov 3;13:1024239. doi: 10.3389/fpls.2022.1024239 (PMC9673822; doi:10.3389/fpls.2022.1024239)
Supplement: Supplementary file 11 [file Table_1.doc]

Supplementary Table S1 GenBank accession numbers /transcript numbers of all sequences for Phylogenetic analysis

| ID/transcript number | species |
| --- | --- |
| Pc_tra25673/f7p0/1704 | *P. chinensis* |
| Pc_tra29672/f11p0/1524 | *P. chinensis* |
| Pc_tra27824/f2p0/1641 | *P. chinensis* |
| Pc_tra29686/f2p0/1549 | *P. chinensis* |
| Pc_tra26704/f5p0/1679 | *P. chinensis* |
| Pc_tra26234/f2p0/1715 | *P. chinensis* |
| Pc_tra28492/f2p0/1604 | *P. chinensis* |
| Pc_tra19941/f2p0/2048 | *P. chinensis* |
| Rsa_AAS55083.1 | *R. sachalinensis* |
| At_OAP11697.1 | *Arabidopsis thaliana* |
| At_Q9ZQ96.1 | *Arabidopsis thaliana* |
| At_OAP09975.1 | *Arabidopsis thaliana* |
| Pc_tra24590/f3p0/1779 | *P. chinensis* |
| Pc_tra26391/f2p0/1752 | *P. chinensis* |
| Pc_tra24574/f7p0/1746 | *P. chinensis* |
| At_OAP14423.1 | *Arabidopsis thaliana* |
| Zm_ONM12917.1 | *Zea mays* |
| At_Q9SY84.1 | *Arabidopsis thaliana* |
| At_Q9ZVX4.1 | *Arabidopsis thaliana* |
| Zm_NP_001147564.1 | *Zea mays* |
| Pc_tra22087/f2p0/1916 | *P. chinensis* |
| At_Q9LXV0.1 | *Arabidopsis thaliana* |
| At_Q9T080.1 | *Arabidopsis thaliana* |
| At_Q9FN26.1 | *Arabidopsis thaliana* |
| At_Q9LPS8.1 | *Arabidopsis thaliana* |
| At_Q9LJA6.1 | *Arabidopsis thaliana* |
| Pc_tra10339/f2p0/2751 | *P. chinensis* |
| Pc_tra29744/f2p0/1541 | *P. chinensis* |
| Pc_tra27534/f3p0/1673 | *P. chinensis* |
| Pc_tra27602/f3p0/1624 | *P. chinensis* |
| Pc_tra32710/f2p0/1379 | *P. chinensis* |
| Dc_XP_020677841.1 | *Dendrobium catenatum* |
| Pc_tra24870/f3p0/1784 | *P. chinensis* |
| Zm_NP_001146271.2 | *Zea mays* |
| At_OAO95244.1 | *Arabidopsis thaliana* |
| At_OAP04815.1 | *Arabidopsis thaliana* |
| Pc_tra17418/f3p0/2174 | *P. chinensis* |
| Rse_Q9AR73.1 | *R. serpentina* |
| Pc_tra28360/f2p0/1592 | *P. chinensis* |
| Pc_tra24635/f2p0/1797 | *P. chinensis* |
| Pc_tra28208/f27p0/1567 | *P. chinensis* |
| At_OAP13096.1 | *Arabidopsis thaliana* |
| At_OAP18465.1 | *Arabidopsis thaliana* |
| Pc_tra24704/f5p0/1777 | *P. chinensis* |
| Pc_tra24513/f4p0/1767 | *P. chinensis* |
| At_Q9FI97.1 | *Arabidopsis thaliana* |
| Dc_XP_020688709.1 | *Dendrobium catenatum* |
| Dc_PKU77367.1 | *Dendrobium catenatum* |
| At_OAP10737.1 | *Arabidopsis thaliana* |
| Dc_PKU65275.1 | *Dendrobium catenatum* |
| Zm_AQK58687.1 | *Zea mays* |
| AS_PKA50344.1 | *Apostasia shenzhenica* |
| At_Q9SGA8.1 | *Arabidopsis thaliana* |
| At_OAO99238.1 | *Arabidopsis thaliana* |
| Pc_tra15728/f2p0/2287 | *P. chinensis* |
| At_OAP16927.1 | *Arabidopsis thaliana* |
| Pc_tra25353/f2p0/1753 | *P. chinensis* |
| At_OAP13716.1 | *Arabidopsis thaliana* |
| Zm_NP_001137065.1 | *Zea mays* |
| As_PKA65436.1 | *Apostasia shenzhenica* |
| Pc_tra24544/f3p0/1718 | *P. chinensis* |
| Pc_tra351/f2p0/5220 | *P. chinensis* |
| Pc_tra16563/f4p0/2237 | *P. chinensis* |
| Pc_tra20290/f2p0/2018 | *P. chinensis* |
| Pc_tra19586/f3p0/2041 | *P. chinensis* |
| Pc_tra6192/f2p0/3220 | *P. chinensis* |
| Pc_tra959/f2p0/4423 | *P. chinensis* |
| Pc_tra31929/f2p0/1436 | *P. chinensis* |
| Pc_tra20085/f5p0/2016 | *P. chinensis* |
| Pc_tra17577/f14p0/2109 | *P. chinensis* |
| Pc_tra18049/f2p0/2142 | *P. chinensis* |
| Pc_tra30764/f2p0/1479 | *P. chinensis* |
| Pc_tra20627/f2p0/1989 | *P. chinensis* |
| Pe_XP_020575676.1 | *P. equestris* |
| Pc_tra23822/f3p0/1850 | *P. chinensis* |
| Pc_tra26864/f5p0/1670 | *P. chinensis* |
| Pc_tra12540/f4p0/2475 | *P. chinensis* |
| Pc_tra6545/f2p0/3169 | *P. chinensis* |
| Pc_tra261/f5p0/5355 | *P. chinensis* |
| Pc_tra5831/f2p0/3252 | *P. chinensis* |
