# Supplementary material for: The gastrodin biosynthetic pathway in Pholidota chinensis Lindl. revealed by transcriptome and metabolome profiling
Source: Front Plant Sci. 2022 Nov 3;13:1024239. doi: 10.3389/fpls.2022.1024239 (PMC9673822; doi:10.3389/fpls.2022.1024239)
Supplement: Supplementary file 12 [file Table_2.doc]

Supplementary Table2 Gene-specific primers used in real-time qPCR

| **Gene** | **Transcript numbers** | **Sequence** |
| --- | --- | --- |
| ADH | 30246/f18p0/1465-F | AGTTGCCCATCTTCCCTCTAAT |
|  | 30246/f18p0/1465-R | GCATCGCTTCGTTCACATAGT |
| HCT | 26704/f5p0/1679-F | GGGCACCGCAATTACTAATACTT |
|  | 26704/f5p0/1679-R | CACATCCACCAACAACCTCTC |
| GT1 | 16563/f4p0/2237-F | TGACCTCGCAACAGATTATGAAC |
|  | 16563/f4p0/2237-R | TTCCAGTGCCTCAACAATAATTCT |
| GT3-01 | 17418/f3p0/2174-F | GTTAATAAGTGGAAGAGCGTAGCA |
|  | 17418/f3p0/2174-R | CACAACCTCCGCATCATCTC |
| GT3-02 | 28360/f2p0/1592-F | GAAGGAGTTGATGGAAGGAGAG |
|  | 28360/f2p0/1592-R | TCACTTAGTAGCCGTTGAATCC |
| GT4 | 261/f5p0/5355-F | TCTGTAACAATCCAATGACCAAGG |
|  | 261/f5p0/5355-R | TCCAACTCTTCGCCTAGTATTCT |
| TUB | TUB | GAATCAACTACCAACCTCCTACAG |
|  | TUB | CTTCCATTCCTTCTCCGACATAC |
